# An in-silico method leads to recognition of hub genes and crucial pathways in survival of patients with breast cancer

**DOI:** 10.1038/s41598-020-76024-2

**Published:** 2020-10-30

**Authors:** Sepideh Dashti, Mohammad Taheri, Soudeh Ghafouri-Fard

**Affiliations:** 1 Department of Medical Genetics, Shahid Beheshti University of Medical Sciences, Tehran, Iran; 2 Urogenital Stem Cell Research Center, Shahid Beheshti University of Medical Sciences, Tehran, Iran

**Keywords:** Cancer, Molecular biology, Systems biology

## Abstract

Breast cancer is a highly heterogeneous disorder characterized by dysregulation of expression of numerous genes and cascades. In the current study, we aim to use a system biology strategy to identify key genes and signaling pathways in breast cancer. We have retrieved data of two microarray datasets (GSE65194 and GSE45827) from the NCBI Gene Expression Omnibus database. R package was used for identification of differentially expressed genes (DEGs), assessment of gene ontology and pathway enrichment evaluation. The DEGs were integrated to construct a protein–protein interaction network. Next, hub genes were recognized using the Cytoscape software and lncRNA–mRNA co-expression analysis was performed to evaluate the potential roles of lncRNAs. Finally, the clinical importance of the obtained genes was assessed using Kaplan–Meier survival analysis. In the present study, 887 DEGs including 730 upregulated and 157 downregulated DEGs were detected between breast cancer and normal samples. By combining the results of functional analysis, MCODE, CytoNCA and CytoHubba 2 hub genes including MAD2L1 and CCNB1 were selected. We also identified 12 lncRNAs with significant correlation with MAD2L1 and CCNB1 genes. According to The Kaplan–Meier plotter database MAD2L1, CCNA2, RAD51-AS1 and LINC01089 have the most prediction potential among all candidate hub genes. Our study offers a framework for recognition of mRNA–lncRNA network in breast cancer and detection of important pathways that could be used as therapeutic targets in this kind of cancer.

## Introduction

Breast cancer is the second most frequent and the fifth cause of cancer-associated mortality^1^. This type of cancer is associated with dysregulation of several genes (including both coding and non-coding ones) and signaling pathways^2^. Breast cancer is a molecularly heterogeneous disorder which is classified to five subtypes including luminal A, luminal B, basal-like, HER2-enriched and normal-like. This classification is based on the presence/ abundance of estrogen receptor (ER), progesterone receptor (PR), HER2 and Ki67^3,4^. However, several recent studies have indicated significance of other genes and signaling pathways in determination of overall survival (OS) of patients^2,5^. Among the recently appreciated genes in this regard are long non-coding RNAs (lncRNAs)^6^. These transcripts are involved in the regulation of fundamental cell survival pathways and have functional interactions with proteins and other non-coding RNAs that participate in the pathogenesis of breast cancer^7^. Identification of such networks is an important step towards design of targeted therapies in breast cancer.

In the current study, we have retrieved data of two microarray datasets (GSE65194 and GSE45827) from the NCBI Gene Expression Omnibus database (GEO). R package was used for identification of differentially expressed genes (DEGs), assessment of gene ontology (GO) and pathway enrichment evaluation. The DEGs were integrated to construct a protein–protein interaction (PPI) network. Next, hub genes were recognized using the Cytoscape software and lncRNA–mRNA co-expression analysis was performed to evaluate the potential roles of lncRNAs.

## Methods

**Figure 1:**
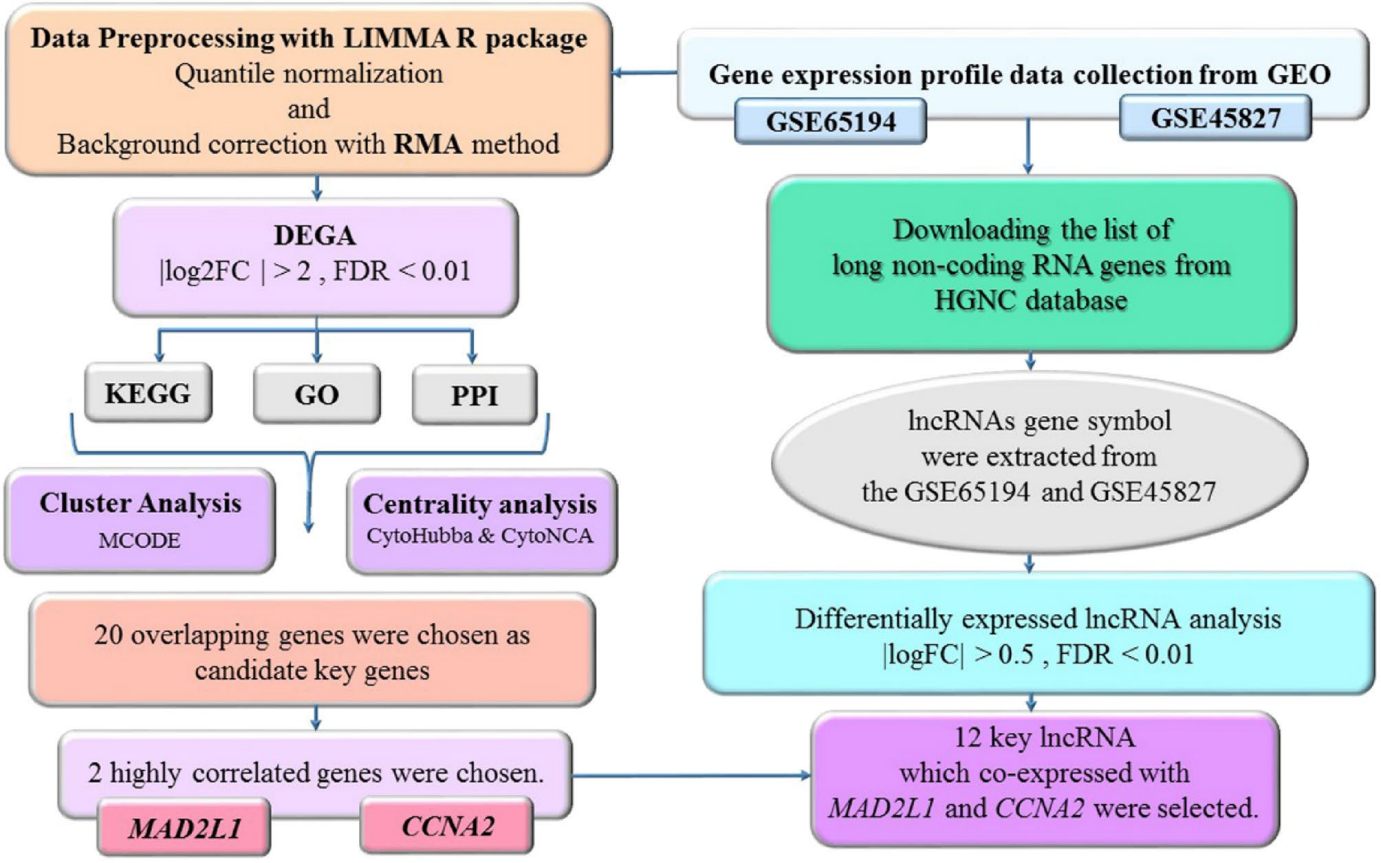
Study design flowchart.

In this study, we used a system biology approach for data mining of two microarray datasets of normal and malignant breast tissue (GSE65194 and GSE45827). We aim to identify differentially expressed genes (DEGs) and lncRNAs and construct an mRNA–lncRNA network based on co-expression analysis. Figure 1 shows summary of the steps accomplished in bioinformatics strategy.

### Gene expression profile data collection

Two gene expression profiles associated with breast cancer (GSE65194 and GSE45827) were obtained from the NCBI Gene Expression Omnibus database (GEO, https://www.ncbi.nlm.nih.gov/geo/). A chip-based platform GPL570 (HG-U133_Plus_2) Affymetrix Human Genome U133 Plus 2.0 Array was applied for both datasets. The GSE65194 included 130 breast cancer samples (41 Triple negative, 30 HER2 positive, 29 Luminal A, 30 Luminal B) and 11 normal breast tissue samples^8^. Similarly, the GSE45827 contained 130 tumor tissue specimen (41 Triple negative, 30 HER2 positive, 29 Luminal A, 30 Luminal B) as well as 11 normal tissues samples^9^.

### Data preprocessing and DEGs identification

All raw data files were subjected to quantile normalization and background correction using Robust Multichip Average (RMA)^10^. RMA is an effective tool in the affy Bioconductor package for both mRNA and lncRNA profiling data^11^. Quality Control assessment was done with AgiMicroRna Bioconductor Package^12^. We conducted a dimensional reduction analysis by performing Principal component analysis (PCA)^13^ with the purpose of finding similarities between each group of samples using ggplot2 package of R software^14^. The linear models for microarray data (LIMMA) R package^15^ in Bioconductor (https://www.bioconductor.org/)^16^ were used to perform differential expression gene analysis (DEGA) between breast cancer and normal breast samples. The Student's t-test was applied and DEGs with false discovery rate (FDR) < 0.01 and a |log2FC (fold change)|> 2 were screened.

### Functional enrichment analysis

To identify the role of DEGs in breast cancer, KEGG Pathway and GO function enrichment analysis in 3 functional ontologies namely biological process (BP), cellular component (CC) and molecular function (MF) were performed using the DAVID system (https://david.ncifcrf.gov/). The adjusted *P* < 0.05 was considered as statistically significant^17^.

### PPI network construction, cluster analysis and key gene identification

To predict interactive relationships among common DEGs encoding proteins, we constructed a PPI network using online STRING database (https://string-db.org/)^18^. The minimum interaction score > 0.4 was required to construct the PPI network. Cytoscape software version 3.7.1 (https://www.cytoscape.org/) was applied to visualize the PPI networks and analyze the hub genes^19^. We used Molecular COmplex DEtection (MCODE) algorithm (version 1.5.1) to find PPI subnetwork and the highly interconnected clusters within the PPI network. MCODE is a Cytoscape plug-in in which we set maximum depth = 100, node score = 0.2, and K-core = 2 as threshold parameters^20^. CytoHubba (version 1.6)^21^ and CytoNCA (version 2.1.6)^22^ are two other plug-in in which provide multiple algorithms to detect hub genes in the network. In addition, identified key genes were selected for additional expression analysis on 1104 cancer and 113 normal samples from the TCGA project in The Encyclopedia of RNA Interactomes (ENCORI) database (https://starbase.sysu.edu.cn/panCancer.php). Pearson correlation coefficient was assessed between hub genes. The correlation coefficients were also checked on TCGA dataset by using Gene Expression Profiling Interactive Analysis (GEPIA) database (https://gepia.cancer-pku.cn/).

### Prediction of lncRNAs function

LncRNA–mRNA co-expression analysis was performed to evaluate the potential roles of lncRNAs. The full list of lncRNA genes with approved HUGO Gene Nomenclature Committee (HGNC) symbols was downloaded from (https://www.genenames.org/)^23^. The list of lncRNA gene names was compared to our dataset gene symbols and overlapped genes were chosen. Then, differentially expressed lncRNAs were selected according to (|logFC|) > 0.5 and the adjusted *P* value < 0.01 cutoff criteria. The reason for application of easier selection criteria was the lower expression level of lncRNAs compared with mRNAs. Then, the Pearson correlation coefficient was calculated between the differentially expressed lncRNA and 2 key protein-coding genes that were obtained from the previous steps based on functional annotation and co-expression analysis (MAD2L1 and CCNA2) in our dataset. LncRNAs with correlation coefficients higher than 0.6 or lower than − 0.6 were chosen as the lncRNAs that co-expressed with MAD2L1 and CCNA2. In order to uncovering the importance of these candidate genes in different molecular subtypes of breast cancer, the expression of these genes was also examined in four breast cancer subtypes, including luminal A, luminal B, basal-like and HER2-enriched**.**

### Survival analysis

Survival analysis was carried out on these candidate hub genes to check out their effects on breast cancer survival. Recurrence free survival (RFS) analysis and overall survival (OS) analysis were performed based on expression data from 6234 breast cancer patients by Kaplan Meier plotter (kmplot.com/) that can evaluate the effect of gene expression on survival in 21 cancer types^24^. We split patients by Mean. In other words, the groups were divided with low expression level and high expression level based on Mean in the survival analysis. The hazard ratio was calculated for both RFS and OS and the *P* value was determined applying log-rank tests.

## Results

### DEGs screening

**Figure 2:**
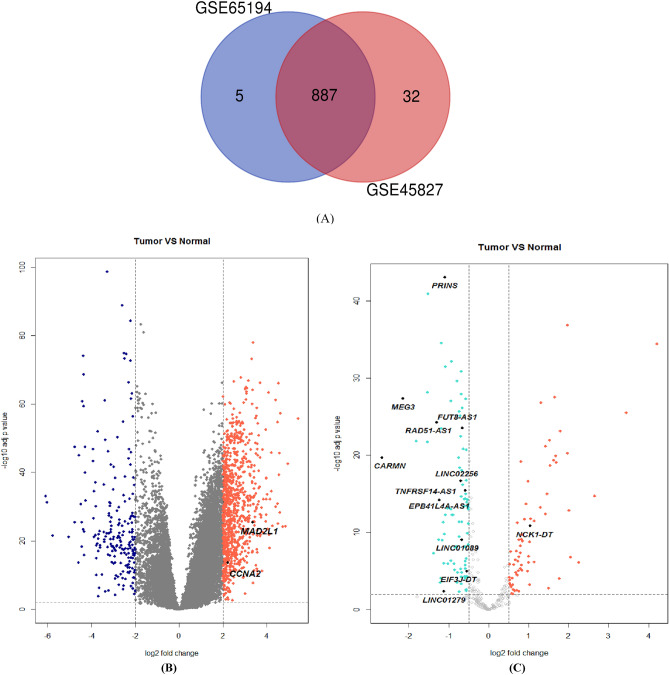
(**a**) A Venn diagram of 887 overlapping DEGs in GSE65194 and GSE45827. (**b**) Volcano plot of significant DEGs with |logFC|> 2. (**c**) Volcano plot of significant differentially expressed lncRNA with |logFC|> 0.5.

Before performing differentially expressed gene analysis (DEGA), background correction and normalization were done and we removed batch effect. We used AgiMicroRna Bioconductor Package for Quality Control assessment. Degradation plots which indicate the quality of RNA hybridization along the probe sets was drawn and the RNA quality was good. Furthermore, box plots for gene expression data were created to assess the distribution of data after normalization. In the box plots the different arrays had the similar median expression level. This result indicated correction was performed properly. Additionally, a PCA plot was drawn to illustrate the spatial distribution of the samples before and after batch effect correction (Supplementary Figure S1a). Principal components analysis (PCA) provides information about the structure of the analyzed dataset. It can be used to find similarities between samples. We found two samples from the normal group which is spatially far from other normal samples. As a consequence, we removed these two samples. Furthermore, a heatmap was drawn to illustrate the correlation between samples using Pheatmap package of R 3.6.1 software^25^ (Supplementary Figure S1b). After correction, removing the batch effects and performing data normalization, 887 DEGs including 730 upregulated and 157 downregulated DEGs were screened between breast cancer and normal samples from GSE65194 and GSE45827 according to |logFC|> 2 and FDR < 0.01 as cut-off criteria. The list of upregulated and downregulated DEGs are indicated in Supplementary Tables S1 and S2, respectively. Figure 2a is a Venn diagram which illustrates the overlap between 2 datasets. Moreover, to visualize the overall gene expression levels of the DEGs, a Volcano plot was created with log2 FC score and log10 *P* values in R software (Fig. 2b).

### KEGG and GO enrichment analysis

**Figure 3:**
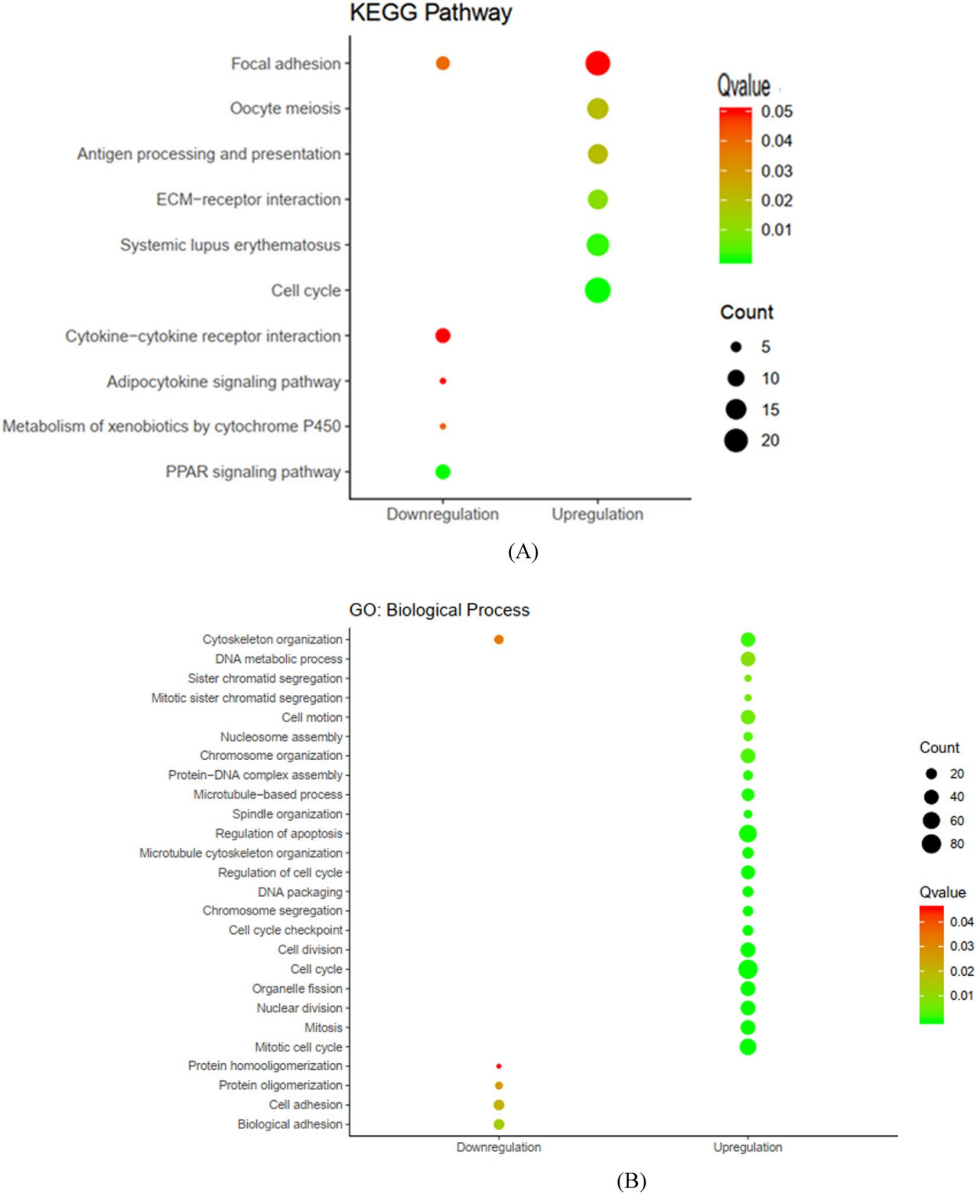

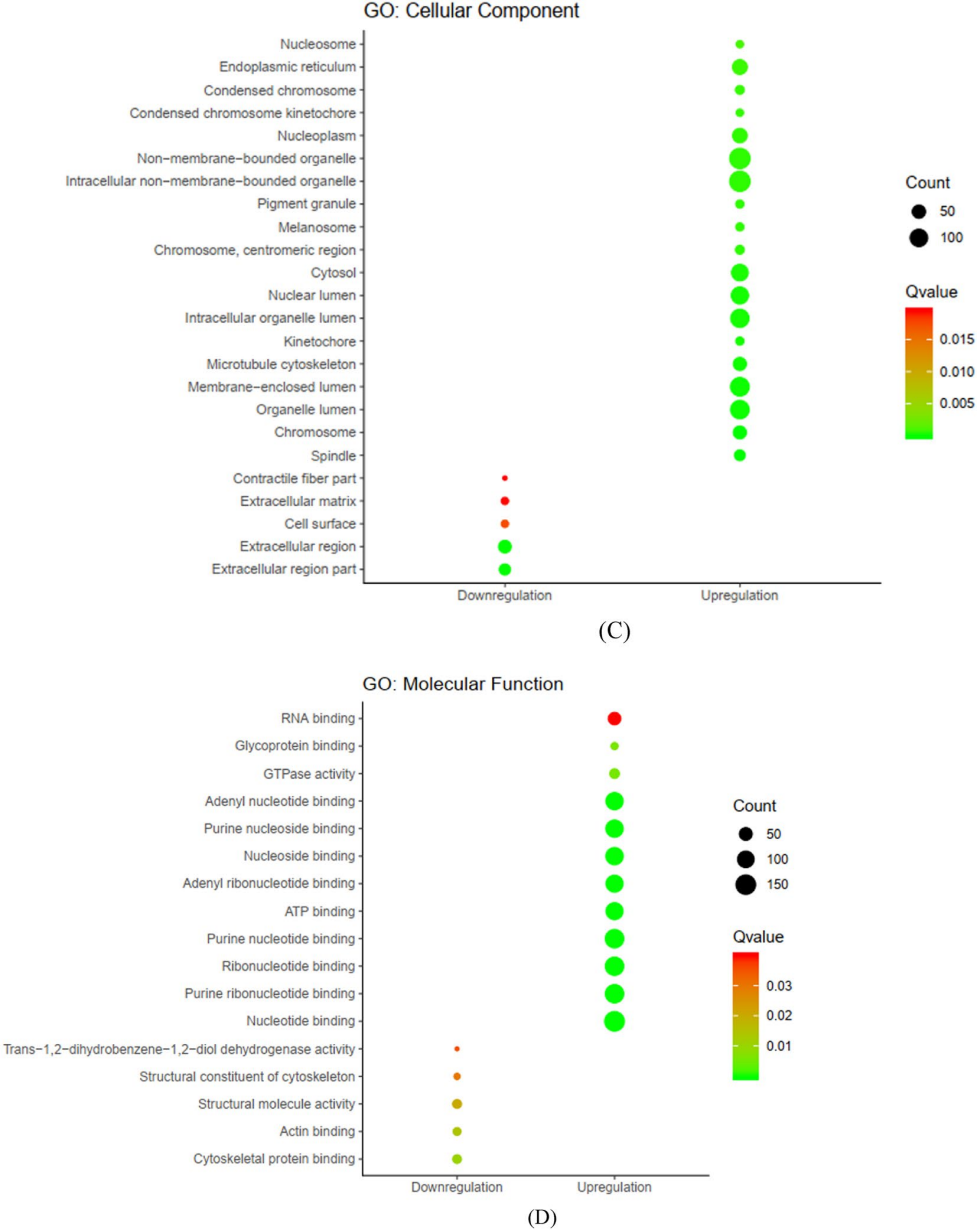
KEGG and GO enrichment analysis. (**a**) KEGG pathways (based on KEGG PATHWAY database^26,27^). (**b**) GO for DEGs, Biological process. (**c**) GO for DEGs, Cellular component. (**d**) GO for DEGs, Molecular function.

To further examine the role of common DEGs in breast cancer, we performed GO and KEGG pathway enrichment analysis^26,27^. We found 10 dysregulated pathways based on the adjusted *P* < 0.05. Up-regulated DEGs were enriched in six pathways including ‘Cell cycle’, ‘Oocyte meiosis’ and ‘Focal adhesion’. Down-regulated DEGs were enriched in five pathways including ‘Peroxisome-proliferator-activated receptors (PPAR) signaling pathway’, ‘Metabolism of xenobiotics by cytochrome P450’, ‘Adipocytokine signaling pathway’ and ‘Cytokine-cytokine receptor interaction’ pathways (Fig. 3a). The results for each GO functional analysis are presented in Fig. 3b–d. The genes enriched in KEGG pathway and GO enrichment analysis have shown in Supplementary Tables S3–S6.

### PPI network construction and module analysis

**Figure 4:**
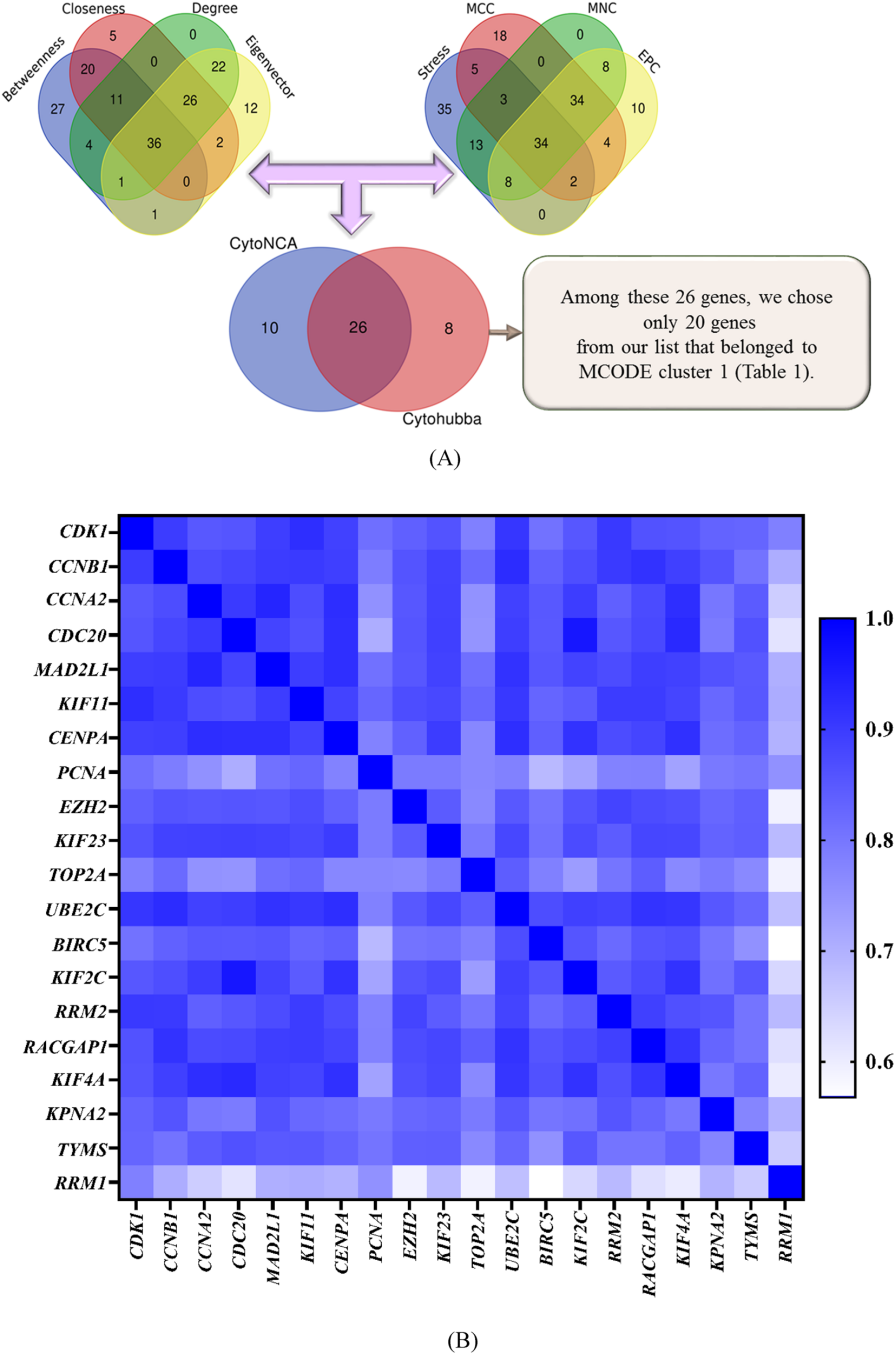
(**a**) A Venn diagram of 26 overlapping genes between different calculation methods of Cytohubba and CytoNCA. (**b**) Heatmap correlation plot for 20 candidate genes (depicted using Pheatmap package of R software^25^).

The interactive information among DEGs and the PPI network was obtained using the STRING online database. Among the total of common DEGs, 887 DEGs (730 up-regulated and 157 down-regulated) were filtered into the PPI network with 887 nodes and 10,398 edges, at a combined score > 0.4. Finally, Genes with a combined score > 0.9 were selected as key DEGs to be imported into Cytoscape. The Cytoscape software was applied to evaluate the interactive relationships between the candidate proteins. Afterward, two clusters consist of 65 nodes and 23 nodes were screened with a cut-off k-score = 12 depend on the MCODE scoring system (Supplementary Figure S2). The CytoNCA and the CytoHubba are two Cytoscape plug-in for centrality analysis and give us some insight into the most influential nodes or edges in a network. We ran CytoHubba application and extracted data from four calculations methods (EPC, MCC, MNC, and Stress). The top 100 nodes ranked by these four methods were selected (Supplementary Table S7). Moreover, four algorithms from CytoNCA application (Degree, Eigenvector, Betweenness, and Closeness) were employed and the top 100 nodes based upon these four approaches were obtained (Supplementary Table S7). Besides, a Venn diagram was created to identify the significant hub genes that are similar between all groups. The result of Venn diagram is mentioned in Supplementary Table S8. Eventually, through overlapping analysis, we identified a list of 26 key genes most of them belonged to MCODE cluster 1 (Supplementary Table S8). Since highly interconnected proteins in a network accumulate in a cluster, we chose only 20 genes from our list that belonged to cluster 1 (Table 1). All the selection steps are illustrated in Fig. 4a.

**Table 1:** Key differentially expressed genes acquired by centrality analysis.

Gene	logFC	adj.*P*.Val	MCODE	Centrality analysis by			CytoNCA	Centrality analysis by			CytoHubba
			MCODE Score	Betweenness	Closeness	Degree	Eigenvector	EPC	MCC	MNC	Stress
CDK1	3.729327	3.03E−28	46.020339	6576.003525	0.551637	131	0.1437205	55.3	9.22E+13	158	356,658
CCNB1	3.115417	1.59E−27	46.020339	3303.18478	0.534146	116	0.1417335	55.9	9.22E+13	133	213,424
CCNA2	2.219921	2.57E−14	46.020339	2534.615585	0.52019	105	0.1342352	53.4	9.22E+13	122	163,390
CDC20	2.756389	1.63E−14	46.020339	2758.724299	0.496036	103	0.1345755	51.8	9.22E+13	116	116,042
MAD2L1	3.346366	4.29E−26	46.020339	1131.260005	0.48079	94	0.1333797	52.9	9.22E+13	110	72,556
KIF11	3.207367	4.39E−28	46.020339	1464.21417	0.487751	92	0.1298102	50.4	9.22E+13	104	95,940
CENPA	2.75168	8.64E−15	47.094949	1704.200531	0.481319	92	0.1282006	50.3	9.22E+13	100	74,860
PCNA	2.453675	1.03E−37	43.857039	2862.973827	0.489385	92	0.1150848	47.9	9.22E+13	106	108,266
EZH2	2.942149	1.48E−27	42.305272	3357.588136	0.511085	91	0.1046336	47.1	9.22E+13	112	243,314
KIF23	2.687969	7.39E−20	46.020339	1657.240201	0.493799	89	0.1254939	49	9.22E+13	97	99,424
TOP2A	4.595822	5.72E−29	46.020339	1122.678983	0.493243	88	0.1293264	51.4	9.22E+13	104	94,808
UBE2C	3.062068	1.20E−22	46.020339	1455.353604	0.466951	88	0.1219277	48.8	9.22E+13	101	67,434
BIRC5	2.792394	5.43E−15	46.020339	1678.399139	0.493799	88	0.1287475	48.9	9.22E+13	99	108,288
KIF2C	2.389307	1.51E−15	46.94026	1182.010234	0.487751	88	0.1259678	48	9.22E+13	97	85,072
RRM2	4.481084	1.30E−31	46.020339	1745.829126	0.497727	86	0.1228496	50.9	9.22E+13	101	110,592
RACGAP1	2.400852	1.87E−23	46.020339	1361.211732	0.493799	86	0.1225891	49.8	9.22E+13	94	93,686
KIF4A	2.206078	2.43E−13	46.94026	1008.406749	0.478689	80	0.1192849	46	9.22E+13	88	65,850
KPNA2	3.428273	1.09E−42	46.880102	961.5679185	0.47454	78	0.111362	46.1	9.22E+13	86	91,340
TYMS	2.878076	1.89E−21	47.774118	1340.60442	0.473514	76	0.1156782	48.1	9.22E+13	92	99,082
RRM1	2.268661	2.13E−25	40.445411	1145.682149	0.45768	69	0.0981711	43.2	9.22E+13	80	69,510

logFC, log2 fold change; adj.*P*.Val, adjusted *P* value; EPC, Edge Percolated Component; MCC, Maximal Clique Centrality; MNC, Maximum Neighborhood Component.

### Key genes functional annotation and co-expression analysis

GO enrichment and KEGG pathway analysis on these 20 genes indicated that four pathways were enriched, including cell cycle, progesterone-mediated oocyte maturation, oocyte meiosis, and p53 signaling pathway. CCNA2, CDK1, MAD2L1, and CCNB1 were significantly enriched in some biological aspects such as cell cycle, mitosis, nuclear division, M phase, cell cycle and progesterone-mediated oocyte maturation pathways. In particular, by checking the expression data of 1104 cancer and 113 normal samples from the TCGA project in ENCORI database, we found that these four genes showed strong expression in the breast cancer specimens as compared to their expression in normal breast tissue ( Including : *MAD2L1,* Fold change: 4.28, Adjusted *P* value: 1.4e−70; *CCNA2,* Fold change: 6.88, Adjusted *P* value: 3.2e−91; *CCNB1,* Fold change: 5.63, Adjusted *P* value: 1.8e−111; *CDK1*, Fold change: 8.54, Adjusted *P* value: 5.3e−121). Additionally, we calculated the Pearson correlation for these 20 candidate genes and found a strong and significant correlation between them (Supplementary Table S9, Fig. 4b). Interestingly, *CCNA2* and *MAD2L1* which are two important genes in the cell cycle pathway and some crucial biological processes related to cell division, were highly correlated genes with a correlation coefficient higher than 0.9 in our analysis. Furthermore, these two genes correlation in TCGA dataset in the GEPIA database was consistent with our analysis.

### Identification of differentially expressed lncRNAs and co-expression analysis

After downloading the list of lncRNA genes from HGNC database, lncRNAs genes symbols were extracted from the GSE65194 and GSE45827. A total of 334 lncRNA probes were identified in these two datasets by using this approach. Finally, 159 lncRNAs probe ID with |logFC|> 0.5 and adjusted *P* value < 0.01 among 20 normal samples and 258 breast tissue samples were picked out (Fig. 2c). Among these lncRNAs, 77 lncRNAs were up-regulated (Supplementary Table S10) and 80 lncRNAs were down-regulated (Supplementary Table S11) in breast cancer. We calculated Pearson correlation coefficient between differentially expressed lncRNAs and MAD2L1 and CCNA2 based on their expression value. LncRNA with Pearson correlation coefficient ≥ 0.6 or ≤  − 0.6 were selected as key lncRNA which co-expressed with MAD2L1 and CCNA2. Totally, 12 lncRNAs meet this criterion (Table 2). Additionally, Table 3 indicates the expression of these genes in four breast cancer subtypes. Our selected genes appear to be more important in more aggressive sub-types (basal-like and HER-enriched). However, deregulation of CARMN, PRINS and MEG3 may be crucial in all subtypes of breast cancer.

**Table 2:** Key lncRNAs which co-expressed with *MAD2L1* and *CCNA2.*

Symbol	*MA2L1*		*CCNA2*	
	Pearson correlation	*P* value	Pearson correlation	*P* value
*MEG3*	− 0.68	< 2.2e−16	− 0.73	< 2.2e−16
LINC01279	− 0.66	< 2.2e−16	− 0.68	< 2.2e−16
*EIF3J-DT*	− 0.64	< 2.2e−16	− 0.68	< 2.2e−16
LINC01089	− 0.64	< 2.2e−16	− 0.67	< 2.2e−16
*RAD51-AS1*	− 0.64	< 2.2e−16	− 0.63	< 2.2e−16
*TNFRSF14-AS1*	− 0.61	< 2.2e−16	− 0.63	< 2.2e−16
LINC02256	− 0.60	< 2.2e−16	− 0.62	< 2.2e−16
*PRINS*	− 0.60	< 2.2e−16	− 0.62	< 2.2e−16
*EPB41L4A-AS1*	− 0.60	< 2.2e−16	− 0.62	< 2.2e−16
*CARMN*	− 0.60	< 2.2e−16	− 0.60	< 2.2e−16
*FUT8-AS1*	− 0.60	< 2.2e−16	− 0.60	< 2.2e−16
*NCK1-DT*	0.65	< 2.2e−16	0.66	< 2.2e−16
*CCNA2*	0.93	< 2.2e−16	1	< 2.2e−16
*MAD2L*1	1	< 2.2e−16	0.936209	< 2.2e−16

**Table 3:** Relative expression of our candidate genes in different molecular subtypes of breast cancer and healthy breast tissue in **GSE65194 and GSE45827.**

Symbol	Basal-like		HER2-enriched		Luminal A		Luminal B	
	logFC	adj.*P*.Val	logFC	adj.*P*.Val	logFC	adj.*P*.Val	logFC	adj.*P*.Val
MAD2L1	4.4	3.02E−61	3.57	6.58E−44	1.709	3.53E−13	3.297	4.45E−37
CCNA2	3.91	9.31E−50	2.78	1.50E−28	0.644	0.00823	1.986	1.09E−15
RAD51-AS1	− 1.6	1.47E−33	− 1.6	1.57E−30	− 0.9	6.18E−12	− 1.27	5.54E−21
LINC01089	− 1	2.44E−18	− 0.8	6.34E−11	− 0.32	0.00964	− 0.71	5.79E−09
EIF3J-DT	− 0.9	1.02E−13	− 0.8	1.66E−10	− 0.04	0.77445	− 0.46	0.00031
LINC02256	− 0.9	1.13E−21	− 0.9	3.56E−22	− 0.44	3.20E−06	− 0.57	1.22E−09
TNFRSF14-AS1	− 0.8	8.62E−26	− 0.8	6.44E−25	− 0.34	6.84E−06	− 0.5	7.35E−11
CARMN	− 3.2	6.13E−22	− 3	4.42E−18	− 1.91	4.53E−08	− 2.41	5.23E−12
EPB41L4A-AS1	− 1.6	1.78E−20	− 1.3	2.99E−13	− 0.7	0.0002	− 1.24	3.19E−11
LINC01279	− 2.1	2.58E−08	− 0.9	0.022916	0.484	0.27445	− 0.66	0.10494
MEG3	− 2.8	7.98E−40	− 2.1	2.33E−25	− 1.55	5.77E−14	− 1.91	8.24E−20
FUT8-AS1	− 0.8	2.61E−31	− 0.8	7.15E−26	− 0.49	5.63E−11	− 0.55	1.80E−13
PRINS	− 1.2	4.18E−44	− 1.2	2.64E−38	− 0.92	4.31E−24	− 1.09	1.10E−31
NCK1-DT	1.58	1.22E−22	0.94	4.64E−09	0.625	0.00022	0.897	8.21E−08

logFC, log2 fold change; adj.*P*.Val, adjusted *P* value.

### Survival analysis of candidate hub genes

Associations between expression of candidate hub genes and RFS and OS of the breast cancer patients were evaluated using KM method to estimate the prognostic importance of the hub genes in our study. The results indicated that low expression of MAD2L1, CCNA2 and NCK1-DT lead to higher RFS rate than high expression. Inversely, high expressions of MEG3, RAD51-AS1, PRINS, LINC01089, LINC02256, FUT8-AS1, LINC01279, CARMN, EPB41L4A-AS1, EIF3J-DT and TNFRSF14-AS1 result in a significantly longer RFS time among patients with breast cancer. The results showed that MAD2L1, CCNA2, RAD51-AS1 and LINC01089 have the most prediction potential based on RFS among all candidate hub genes. Besides, hazard ratio was also calculated for OS. High expression of MAD2L1, CCNA2 and FUT8-AS1 lead to lower OS rate than low expression. On the other hand, low expressions of LINC01279, RAD51-AS1 and CARMN were correlated with significantly worse OS in breast cancer patients. Other candidate hub genes expressions were not significantly relevant to OS (Table 4).

**Table 4:** Recurrence free survival (RFS) and overall survival (OS) of candidate hub genes.

Gene name		Multivariate analysis for RFS			Univariate analysis for RFS			Multivariate analysis for OS			Univariate analysis for OS		
Probe ID	Symbole	HR	CI	logrank *P*	HR	CI	logrank *P*	HR	CI	logrank *P*	HR	CI	logrank *P*
210794_s_at	*MEG3*	0.8	0.71–0.89	0.0001	0.73	0.65–0.81	1.30e−08	0.93	0.74–1.17	0.5396	0.82	0.66–1.02	0.0713
227061_at	*LINC01279*	0.74	0.63–0.87	0.0003	0.69	0.59–0.81	4.2e−06	0.73	0.53–1.01	0.0553	0.72	0.53–0.99	0.0391
235124_at	*EIF3J-DT*	0.57	0.48–0.68	0	0.5	0.42–0.58	< 1e−16	0.76	0.54–1.07	0.1189	0.62	0.45–0.85	0.0029
226369_at	*LINC01089*	0.5	0.42–0.58	0	0.46	0.39–0.54	< 1e−16	0.84	0.6–1.17	0.2993	0.72	0.53–0.99	0.0433
1560081_at	*RAD51-AS1*	0.45	0.38–0.53	0	0.41	0.35–0.49	< 1e−16	0.7	0.5–0.99	0.0427	0.61	0.45–0.84	0.0023
232190_x_at	*TNFRSF14-AS1*	0.63	0.54–0.74	0	0.58	0.5–0.68	1.4e−11	0.86	0.62–1.18	0.3494	0.76	0.55–1.04	0.0868
234423_x_at	*LINC02256*	0.63	0.53–0.74	0	0.58	0.49–0.68	5.2e−12	1.26	0.91–1.75	0.1708	1.06	0.78–1.46	0.6976
216051_x_at	*PRINS*	0.7	0.63–0.78	0	0.69	0.62–0.77	2.4e−11	0.91	0.73–1.13	0.3928	0.86	0.69–1.06	0.1658
225698_at	*EPB41L4A-AS1*	0.77	0.65–0.91	0.0023	0.65	0.55–0.76	3.3e−08	0.91	0.65–1.28	0.5966	0.75	0.55–1.03	0.0781
1558828_s_at	*CARMN*	0.62	0.53–0.73	0	0.59	0.51–0.69	5.3e−11	0.61	0.44–0.84	0.0028	0.56	0.41–0.77	0.0003
242889_x_at	*FUT8-AS1*	0.8	0.68–0.93	0.0046	0.75	0.64–0.88	0.00028	1.74	1.25–2.43	0.0011	1.54	1.11–2.12	0.0083
228799_at	*NCK1-DT*	1.06	0.91–1.25	0.4498	1.18	1.01–1.38	0.035	0.95	0.68–1.32	0.751	1.09	0.8–1.49	0.5982
203418_at	*CCNA2*	1.24	1.09–1.42	0.0011	1.84	1.64–2.05	< 1e−16	1.36	1.05–1.77	0.0193	1.55	1.25–1.93	5.0e−05
203362_s_at	*MAD2L1*	1.66	1.47–1.87	0	1.86	1.67–2.08	< 1e−16	1.8	1.42–2.28	0	2.02	1.62–2.51	1.8e−10

HR, hazard ratio; CI, confidence interval; RFS, recurrence free survival; OS, overall survival.

A multivariate analysis was performed for MKI67, ESR1, and HER2 (ERBB2).

## Discussion

In the present study, we used a bioinformatics strategy to identify key genes and signaling pathways in breast cancer pathogenesis with a focus on the role of lncRNAs and their interactions with protein-coding genes. Such interactions can be assessed using experimental approaches which are costly and laborious. Bioinformatics methods for such purpose fall into two groups: strategies that use sequence, structural data and physicochemical features, and methods that are based on network construction. The latter can provide the inherent characteristics of topological configuration of associated biological networks which is often disregarded by the former strategies^6^. In the present work, we used GPL570 which is a good platform to evaluate the expression level of lncRNAs in tumorigenesis^11,28^. We identified 730 upregulated and 157 downregulated DEGs between breast cancer and normal samples. Up-regulated DEGs were enriched in ‘Cell cycle’, ‘Oocyte meiosis’ and ‘Focal adhesion’. A previous bioinformatics study using topological characteristics of genes in breast cancer has identified these pathways as hub subnetworks^29^. The role of these pathways has been acknowledged in the pathogenesis of another hormone related cancer namely prostate cancer^30^. We also detected down-regulated DEGs were enriched in ‘PPAR signaling pathway’, ‘Metabolism of xenobiotics by cytochrome P450′, ‘Adipocytokine signaling pathway’ and ‘Cytokine-cytokine receptor interaction’ pathways. PPARs are nuclear hormone receptors which participate in modulation of different aspects of tumorigenesis such as cell proliferation, survival and apoptosis^31^. Xenobiotic metabolizing enzymes are also involved in the tumorigenesis and response of cancer patients to therapeutic options. Integration of expression data of these genes with eQTL data and allele frequency data from the 1000 Genomes project has shown considerable inter-population differences in the related pathways which might influence cancer prognosis and response to treatment^32^. Adipocytokines can also influence cell proliferation and survival, and malignant phenotypes of breast cancer cells through regulation several cellular and molecular pathways thus aggravating survival of patients^33^. Cytokine signaling has important functions in formation, proliferation, and migration of breast cancer, thus modulating invasiveness, angiogenesis and metastatic potential of these cells^34^.

Our in silico analyses revealed that *CCNA2, CDK1, MAD2L1* and *CCNB1* were significantly enriched in several biological pathways. These four genes showed strong expression in breast cancer samples as compared to their expression in normal breast tissue. Notably, these four genes have been among the top dysregulated genes in small cell lung cancer as revealed by GO, KEGG analysis and construction of PPI network^35^. Such similarity between these two different types of cancers implies fundamental role of these genes in the carcinogenesis process and potentiates them as therapeutic targets. MAD2L1 form a complex with the APC/C and CDC20 and subsequently stimulate the M-A checkpoint to halt the transition of cell at this stage in the presence of anomalous segregation of chromatin. Yet, over-expression of E2F1 in atypical cells affects the formation of the mentioned complex leading to cell cycle transition even in the presence of abnormal chromosomes^36^. CDK1/cyclin B is a maturation-promoting factor^37^ and the checkpoint for G2/M transition^38,39^, so it is expected to be involved in the process of cell cycle regulation and tumorigenesis. We also identified 12 lncRNAs with significant correlation with MAD2L1 and CCNB1 genes. As expected from KEGG analysis, KM analysis indicated that low expression of MAD2L1, CCNA2 and NCK1-DT lead to higher RFS rate than high expression. Inversely, high expressions of MEG3, RAD51-AS1, PRINS, LINC01089, LINC02256, FUT8-AS1, LINC01279, CARMN, EPB41L4A-AS1, EIF3J-DT and TNFRSF14-AS1 result in a significantly longer RFS time among patients with breast cancer. Additionally, hazard ratio was also calculated for OS. High expression of MAD2L1, CCNA2 and FUT8-AS1 and low expressions of LINC01279, RAD51-AS1 and CARMN were correlated with significantly worse OS in breast cancer patients, while Other candidate hub genes expression were not significantly relevant to OS.

According to previous studies MEG3 is down-regulated in breast cancer tissues^40–42^. Recently, Zhang et al. showed MEG3 ability in promoting breast cancer growth and induction of apoptosis by activating ER stress, NF-κB and p53 pathways in breast cancer cell line^43^. RAD51-AS1, also known as TODRA is transcribed from upstream of *RAD51* in a divergent manner. Gazy et al. identified a conserved E2F1 binding site in the promoter region of RAD51-AS1 and considered this lncRNA as a target gene of E2F1 in breast cancer. RAD51-AS1 negatively regulates RAD51 expression and higher expression of RAD51-AS1 has been associated with a less aggressive tumor phenotype^44^. PRINS (Psoriasis susceptibility-related RNA Gene Induced by Stress) is a stress induced lncRNA which regulates apoptosis^45,46^. Min Yu et al. considered PRINS as a HIF-1α dependent lncRNA due to its significant over-expression in hypoxic conditions in renal tubular cells^47^. Moreover, increased levels of PRINS have been observed in colorectal adenocarcinoma cells. This lncRNA interacts with trefoil factor 3 (TFF3), AKT/PI3K signaling pathway and miR-491-5p^48^. PRINS levels were down-regulated in MCF-7 and MDA-MB-231 cell lines following exposure to the apoptotic and anti-proliferative agent CCT137690^49^. LINC01089 (also known as LncRNA Inhibiting Metastasis; LIMT) is an EGF regulated lncRNA which is down-regulated in breast cancer tissues and cell lines, especially in aggressive subtypes of breast cancer^50^. Yuan et al. have reported a significant correlation between low expression of LINC01089 and lymph node metastasis and poor prognosis of breast cancer. LINC01089 is modulates breast tumorigenesis by inhibiting β-catenin transcription and consequently blocking Wnt/β-catenin signaling^51^. LINC02256 (ENSG00000261064) is a validated novel long intergenic non-protein coding RNA with 2 transcripts which is located on 15q13.3. Based on GTEx (Release v6) results, it has ubiquitous expression in breast and other tissues. Potential contribution of this lncRNA in breast cancer should be evaluated in future studies. FUT8‐AS1 was up-regulated in endometrioid endometrial cancer patients in association with poor survival^52^. According to another TCGA data mining study on glioblastoma, FUT8-AS1 *over-expression has been associated with* poor patients outcomes^53^. LINC01279 was significantly upregulated in patients with endometriosis. Based on Liu et al. study, there is a strong association between this lncRNA and cell cycle-dependent kinase-14 and CXC motif chemokine ligand-12. Hence, LINC01279 might contribute in the pathogenesis of endometriosis^54^. In another bioinformatics analysis of differential gene expression in breast cancer LINC01279 was significantly down-regulated^55^. However, further studies should be done to elucidate its function in breast cancer. CARMN (also known as MiR143HG) is recognized as a tumor suppressor in bladder cancer. Xie et al. observed down-regulation of CARMN in bladder cancer tissues compared with normal tissues. Moreover, there was an association between CARMN over-expression and a high survival rate in bladder cancer patients. CARMN/miR‐1275/AXIN2 axis takes part in bladder tumorigenesis by interacting with the Wnt/β‐catenin pathway^56^. Furthermore, there is an association between down-regulation of CARMN and poor survival in endometrial carcinoma^57^. CARMN was significantly down-regulated in hepatocellular carcinoma (HCC) tissues and cells. Over-expression of CARMN was associated with good prognosis. Generally, this gene contributes to development and progression of HCC by blocking the MAPK and Wnt signaling pathways^58^. EPB41L4A-AS1 (also known as TIGA1) is a p53-regulated gene. EPB41L4A-AS1 was down-regulated in many human cancers in correlation with poor prognosis. EPB41L4A-AS1 acts as a repressor of the Warburg effect and is involved in cancer metabolic reprogramming^59^. In a recent study in early stage breast cancer, significant down-regulation of EPB41L4A-AS1 was observed in tumor tissues^60^. EIF3J-DT is a novel lncRNA with no publication reporting its biological function in breast cancer up to now. Based on one study in HCC, EIF3J-DT might have potential prognostic value^61^. In addition, EIF3J-DT regulates multi-drug resistance by interacting with autophagy in gastric cancer^62^. Based on He et al. study, TNFRSF14-AS1 might have a prognostic value in breast cancer but this result needs to further confirmation^63^. Based on the available literature, the identified lncRNAs in the current study has putative roles in the pathogenesis of breast cancer and other types of cancer.

Taken together, in the present study, we intended to introduce a precise method to discover and prioritize the most probable candidate genes involved in breast cancer. Gene expression analysis in different molecular subtypes indicated the importance of our chosen genes in more aggressive subtypes. On the other hand, CARMN, PRINS and MEG3 probably have an important role in pathogenesis of all subtypes of breast cancer. We also added several evidences from literature regarding the role of candidate genes in the pathogenesis of cancer. Although this study provides some impressive evidence for future differential expression studies in breast cancer, the limitation of this study is lack of experimental evaluation of the candidate genes. our in silico method identified a number of hub genes and related lncRNAs which are possibly involved in the pathogenesis of breast cancer and patients' prognosis, so can be used as therapeutic targets or biomarkers for this malignancy.

## Supplementary information

Supplementary Information 1.

Supplementary Information 2.

Supplementary Information 3.

Supplementary Information 4.

Supplementary Information 5.

Supplementary Information 6.

Supplementary Information 7.

Supplementary Information 8.

Supplementary Information 9.

Supplementary Information 10.

Supplementary Information 11.

Supplementary Information 12.
