# Supplementary material for: An in-silico method leads to recognition of hub genes and crucial pathways in survival of patients with breast cancer
Source: Sci Rep. 2020 Oct 30;10:18770. doi: 10.1038/s41598-020-76024-2 (PMC7603345; doi:10.1038/s41598-020-76024-2)
Supplement: Supplementary file 1 — Supplementary Information 1. [file 41598_2020_76024_MOESM1_ESM.docx]

An in-silico method leads to recognition of hub genes and crucial pathways in survival of patients with breast cancer

Sepideh Dashti^1^, Mohammad Taheri^2^, Soudeh Ghafouri-Fard^1^*

1. Department of Medical Genetics, Shahid Beheshti University of Medical Sciences, Tehran, Iran
2. Urogenital Stem Cell Research Center, Shahid Beheshti University of Medical Sciences, Tehran, Iran

**Supplementary figures:**

**Fig S1. (a)** Principal component analysis (PCA) due to exploring the pattern of samples enrichment. GSM1116172 (a normal breast sample) was removed from further analysis in order to its wrong spatial enrichment. **(b)** Heatmap of DEGs for indicating the correlation between samples.


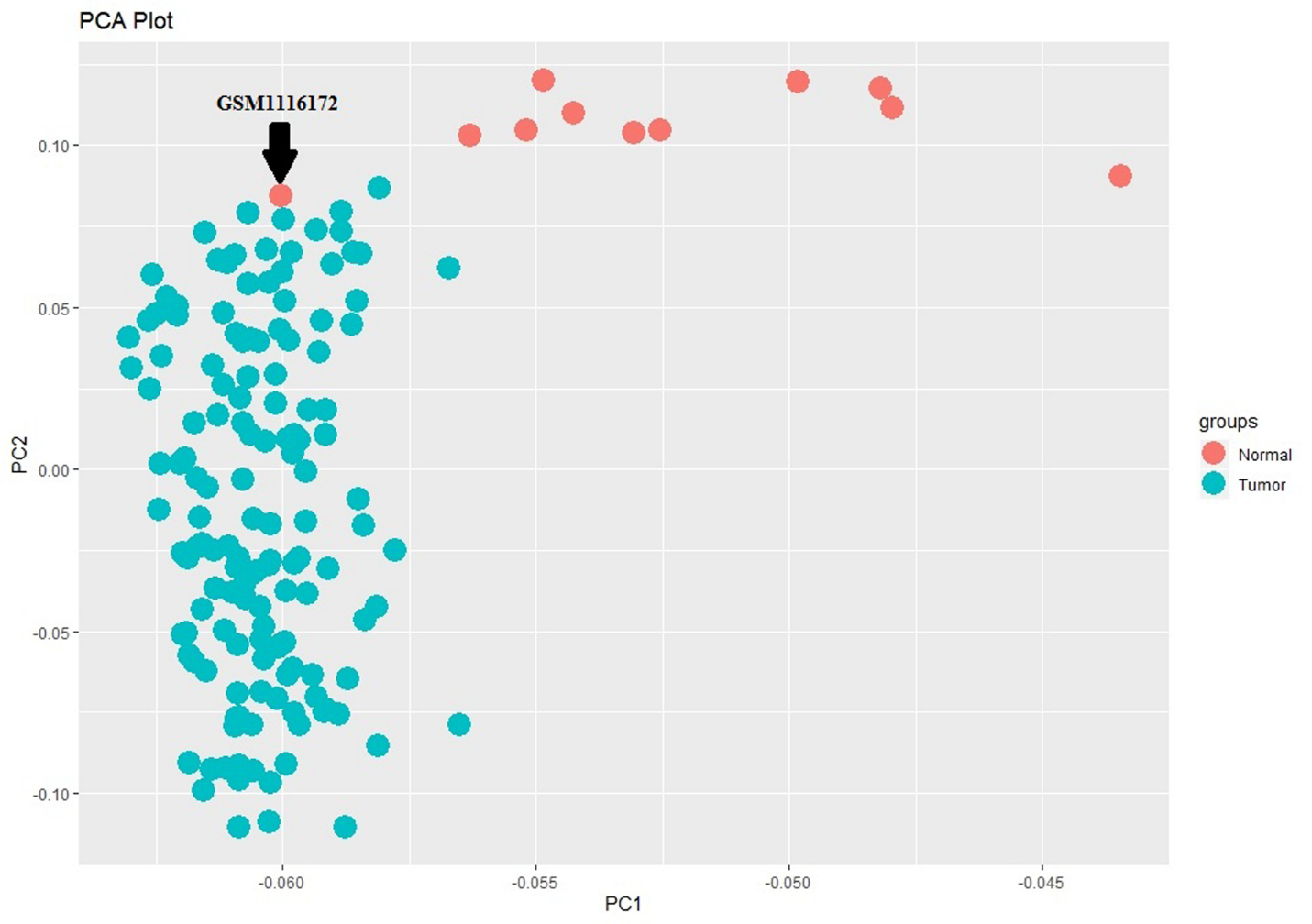


(a)


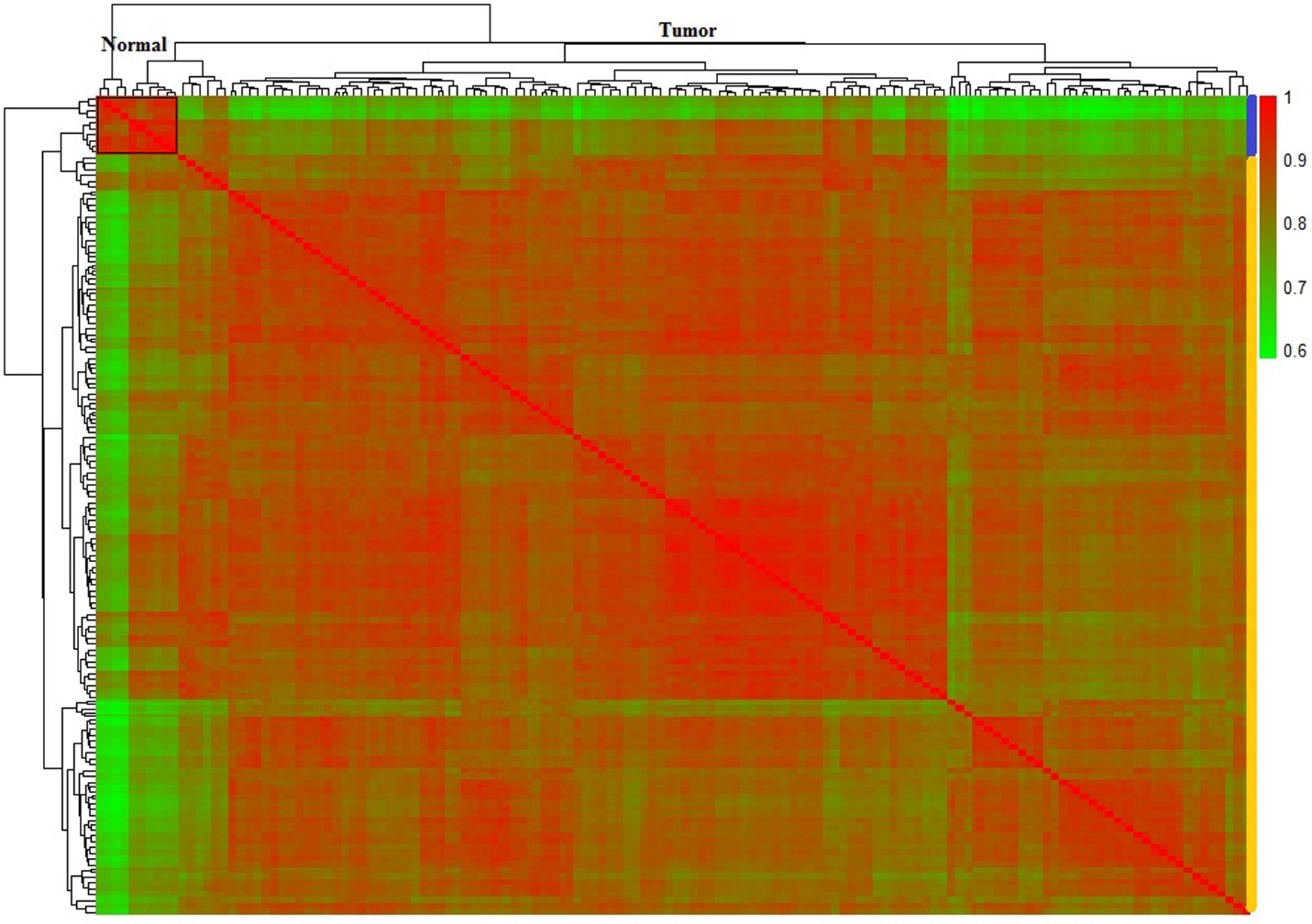


(b)

**Fig S2.** PPI Network Construction and Module analysis**. (a)** Cluster 1 containing 65 nodes and 1923 edges **(b)** cluster 2 containing 23 nodes and 206 edges. These 2 clusters had a cut-off k-score=12 depend on the MCODE scoring system.


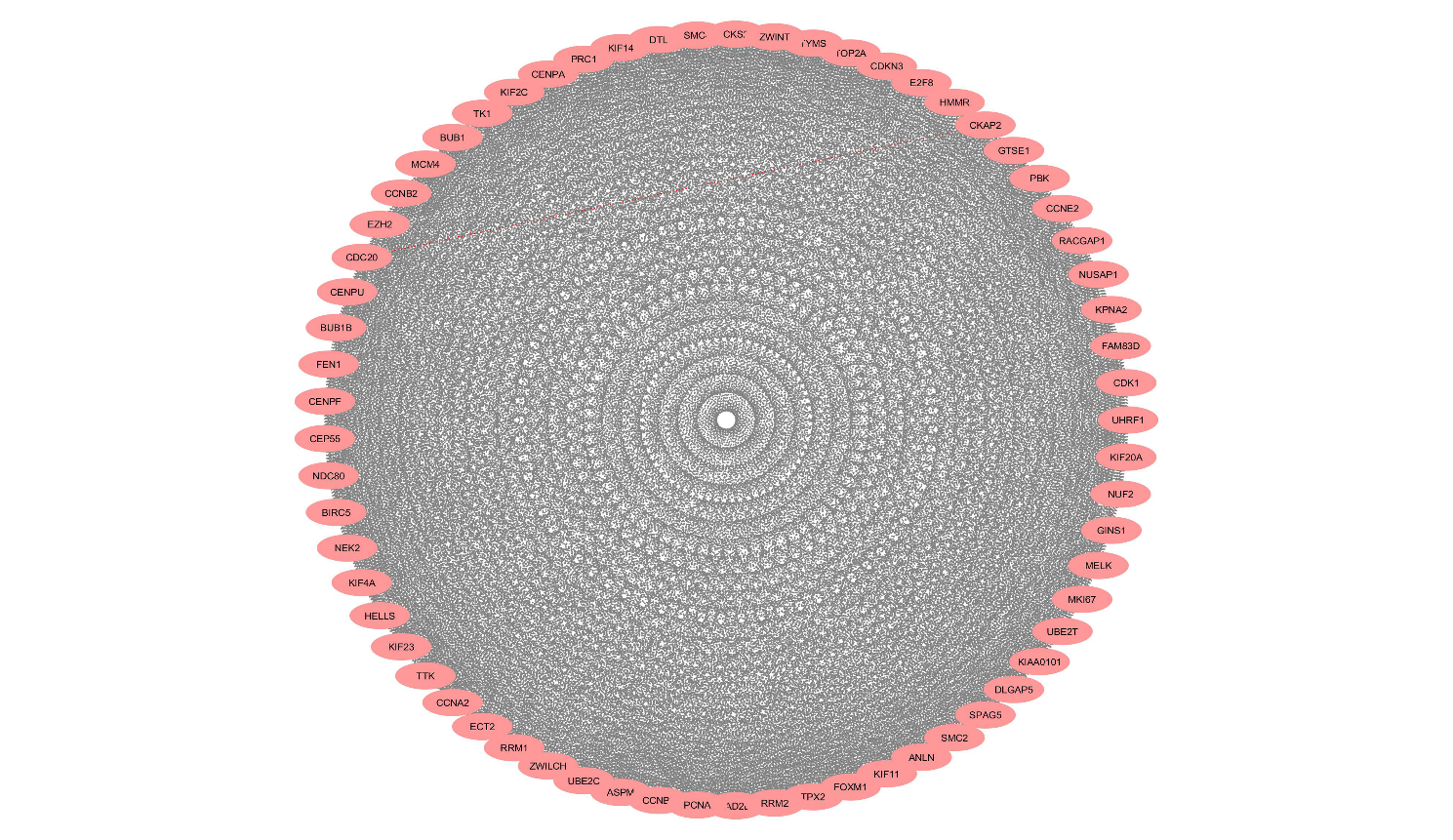


(a)


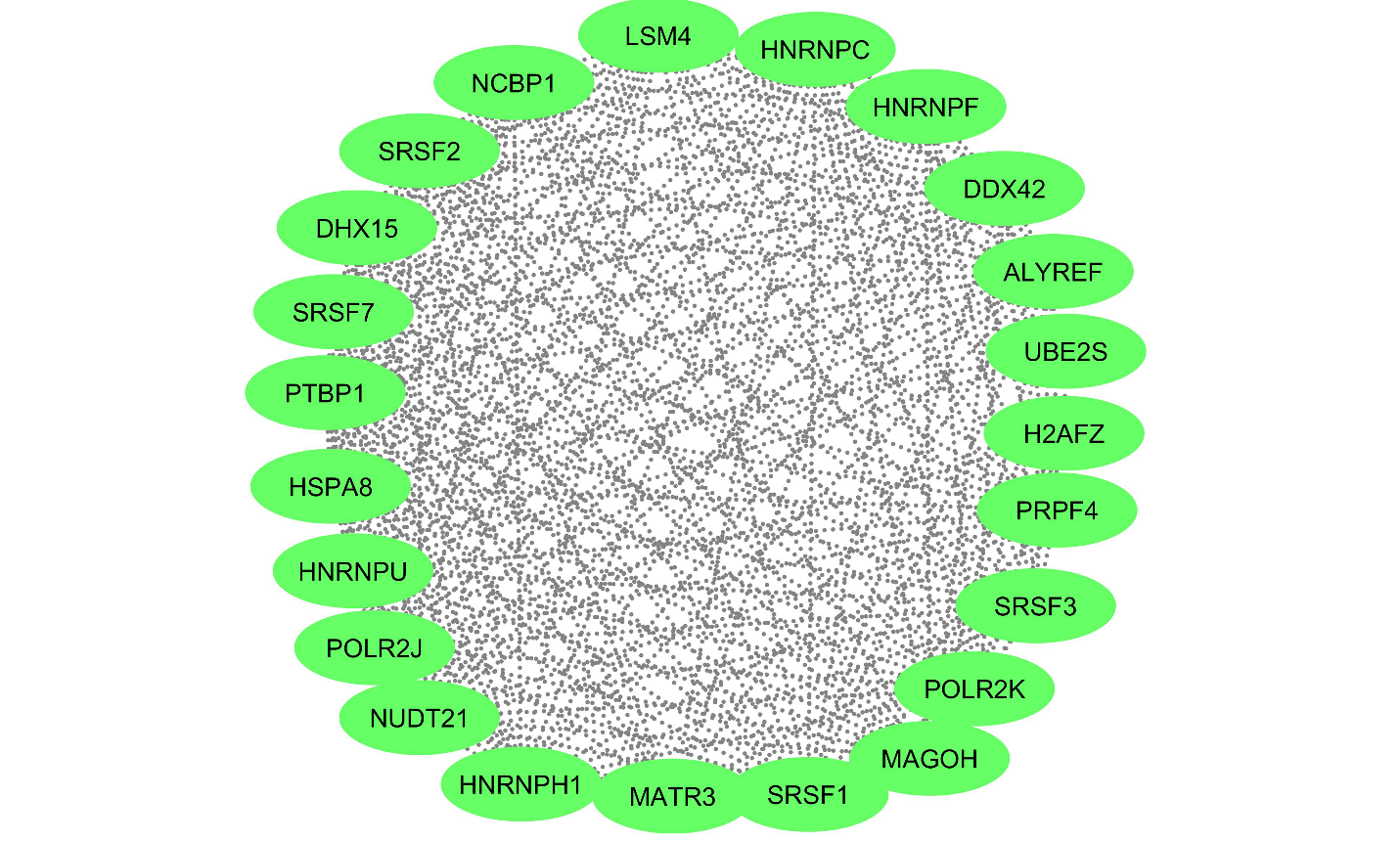


(b)
