# Supplementary material for: An in-silico method leads to recognition of hub genes and crucial pathways in survival of patients with breast cancer
Source: Sci Rep. 2020 Oct 30;10:18770. doi: 10.1038/s41598-020-76024-2 (PMC7603345; doi:10.1038/s41598-020-76024-2)
Supplement: Supplementary file 4 — Supplementary Information 4. [file 41598_2020_76024_MOESM4_ESM.docx]

An in-silico method leads to recognition of hub genes and crucial pathways in survival of patients with breast cancer

Sepideh Dashti^1^, Mohammad Taheri^2^, Soudeh Ghafouri-Fard^1^*

1. Department of Medical Genetics, Shahid Beheshti University of Medical Sciences, Tehran, Iran
2. Urogenital Stem Cell Research Center, Shahid Beheshti University of Medical Sciences, Tehran, Iran

Table S3. The result of KEGG pathway analysis.

| **Category** | **Term** | **SampleGroup** | **P value** | **Count** | **Genes** |
| --- | --- | --- | --- | --- | --- |
| KEGG_PATHWAY | hsa04110:  Cell cycle | Upregulation | 1.40E-07 | 24 | *CDC6, CDK1, YWHAZ, ANAPC5, DBF4, SMAD4, PRKDC, TTK, CDC20, YWHAE, MCM4, SMC3, CCNE2, CCNB1, RAD21, CCNB2, YWHAH, MAD2L1, PCNA, BUB1, BUB1B, GADD45B, CCNA2, BUB3* |
| KEGG_PATHWAY | hsa05322:  Systemic lupus erythematosus | Upregulation | 1.66E-05 | 18 | *HLA-DQB1, HIST2H2AA3, HIST1H2BD, HIST1H2BF, HIST1H2BG, HIST1H2BH, C1S, HLA-DQA1, CD86, HIST1H2BK, H2AFV, HIST2H2BE, HIST1H2BI, FCGR1A, H2AFZ, H2AFY, HIST1H3D, HLA-DPA1, FCGR3B, HLA-DRA* |
| KEGG_PATHWAY | hsa04612:  Antigen processing and presentation | Upregulation | 4.28E-04 | 14 | *HSP90AB1, HLA-DQB1, CREB1, IFI30, NFYB, HSPA1B, CTSS, CANX, HLA-DQA1, HSPA4, HLA-DPA1, CTSB, HSPA8, HLA-DRA* |
| KEGG_PATHWAY | hsa04512:  ECM-receptor interaction | Upregulation | 4.82E-04 | 14 | *SDC1, CD44, COMP, COL3A1, COL1A2, COL1A1, THBS1, COL11A1, ITGB1, COL5A2, COL5A1, HMMR, SPP1, FN1* |
| KEGG_PATHWAY | hsa04114:  Oocyte meiosis | Upregulation | 7.28E-04 | 16 | *CDK1, YWHAZ, ANAPC5, PPP2R5C, AURKA, CDC20, PPP1CB, YWHAE, SMC3, CCNE2, CCNB1, MAPK1, CCNB2, YWHAH, MAD2L1, BUB1* |
| KEGG_PATHWAY | hsa04510:  Focal adhesion | Upregulation | 0.0024531 | 22 | *ACTB, VAV3, ERBB2, COL3A1, PPP1CB, ITGB1, COL5A2, COL5A1, CDC42, MAPK1, PAK2, COMP, VEGFA, RAC1, COL1A2, RHOA, PIK3CA, COL1A1, THBS1, COL11A1, FN1, SPP1* |
| KEGG_PATHWAY | hsa03320:  PPAR signaling pathway | Downregulation | 2.57E-05 | 8 | *LPL, CD36, SORBS1, PLIN1, FABP4, SCD5, ADIPOQ, ANGPTL4* |
| KEGG_PATHWAY | hsa00980:  Metabolism of xenobiotics by cytochrome P450 | Downregulation | 0.04 | 4 | *AKR1C3, AKR1C2, ADH1B, AKR1C1* |
| KEGG_PATHWAY | hsa04510:  Focal adhesion | Downregulation | 0.04 | 7 | *VWF, CAV1, LAMB3, TNXB, TNXA, IGF1, MYLK, PARVA* |
| KEGG_PATHWAY | hsa04060:  Cytokine-cytokine receptor interaction | Downregulation | 0.05 | 8 | *CCL14, CXCL14, LEPR, LIFR, KIT, CX3CL1, CCL28, GHR* |
| KEGG_PATHWAY | hsa04920:  Adipocytokine signaling pathway | Downregulation | 0.05 | 4 | *CD36, LEPR, ACACB, ADIPOQ* |
