# Supplementary material for: An in-silico method leads to recognition of hub genes and crucial pathways in survival of patients with breast cancer
Source: Sci Rep. 2020 Oct 30;10:18770. doi: 10.1038/s41598-020-76024-2 (PMC7603345; doi:10.1038/s41598-020-76024-2)
Supplement: Supplementary file 5 — Supplementary Information 5. [file 41598_2020_76024_MOESM5_ESM.docx]

An in-silico method leads to recognition of hub genes and crucial pathways in survival of patients with breast cancer

Sepideh Dashti^1^, Mohammad Taheri^2^, Soudeh Ghafouri-Fard^1^*

1. Department of Medical Genetics, Shahid Beheshti University of Medical Sciences, Tehran, Iran
2. Urogenital Stem Cell Research Center, Shahid Beheshti University of Medical Sciences, Tehran, Iran

Table S4. The result of GO enrichment analysis (GO for DEGs, Biological process).

| **Category** | **Term** | **SampleGroup** | **Q-value** | **Count** | **Genes** |
| --- | --- | --- | --- | --- | --- |
| GOTERM_BP_FAT | GO:0000278~  mitotic cell cycle | Upregulation | 3.05E-13 | 56 | *KIF23, PRC1, DBF4, TTK, AURKA, GTSE1, FAM83D, KIF2C, RAD21, CCNA2, ASPM, CDK1, CDC6, KIF11, ANAPC5, TPX2, NUSAP1, PBK, UBE2C, PPP1CB, EML4, INHBA, MAD2L1, SPAG5, ZWINT, BUB1B, MAPRE1, KPNA2, NEK2, ANLN, CEP55, ITGB1, CCNG2, PSMB4, CENPA, BUB1, ZWILCH, BUB3, HELLS, CKAP5, DLGAP5, NUF2, CENPF, BIRC5, NDC80, CDC20, CDKN3, SMC2, SMC3, SMC4, CCNB1, CCNB2, NOLC1, GSPT1, PSMD12, PSMD11* |
| GOTERM_BP_FAT | GO:0000280~  nuclear division | Upregulation | 6.10E-13 | 42 | *KIF23, NEK2, ANLN, AURKA, CEP55, CCNG2, FAM83D, KIF2C, RAD21, BUB1, ZWILCH, CCNA2, BUB3, HELLS, ASPM, CDK1, CDC6, KIF11, ANAPC5, CKAP5, DLGAP5, TPX2, NUF2, CENPF, NUSAP1, CDC20, BIRC5, NDC80, PBK, UBE2C, SMC2, SMC3, SMC4, EML4, CCNB1, MAD2L1, CCNB2, NOLC1, SPAG5, ZWINT, BUB1B, MAPRE1* |
| GOTERM_BP_FAT | GO:0007067~  mitosis | Upregulation | 6.10E-13 | 42 | *KIF23, NEK2, ANLN, AURKA, CEP55, CCNG2, FAM83D, KIF2C, RAD21, BUB1, ZWILCH, CCNA2, BUB3, HELLS, ASPM, CDK1, CDC6, KIF11, ANAPC5, CKAP5, DLGAP5, TPX2, NUF2, CENPF, NUSAP1, CDC20, BIRC5, NDC80, PBK, UBE2C, SMC2, SMC3, SMC4, EML4, CCNB1, MAD2L1, CCNB2, NOLC1, SPAG5, ZWINT, BUB1B, MAPRE1* |
| GOTERM_BP_FAT | GO:0000087~  M phase of mitotic cell cycle | Upregulation | 7.12E-13 | 42 | *KIF23, NEK2, ANLN, AURKA, CEP55, CCNG2, FAM83D, KIF2C, RAD21, BUB1, ZWILCH, CCNA2, BUB3, HELLS, ASPM, CDK1, CDC6, KIF11, ANAPC5, CKAP5, DLGAP5, TPX2, NUF2, CENPF, NUSAP1, CDC20, BIRC5, NDC80, PBK, UBE2C, SMC2, SMC3, SMC4, EML4, CCNB1, MAD2L1, CCNB2, NOLC1, SPAG5, ZWINT, BUB1B, MAPRE1* |
| GOTERM_BP_FAT | GO:0048285~  organelle fission | Upregulation | 1.22E-12 | 42 | *KIF23, NEK2, ANLN, AURKA, CEP55, CCNG2, FAM83D, KIF2C, RAD21, BUB1, ZWILCH, CCNA2, BUB3, HELLS, ASPM, CDK1, CDC6, KIF11, ANAPC5, CKAP5, DLGAP5, TPX2, NUF2, CENPF, NUSAP1, CDC20, BIRC5, NDC80, PBK, UBE2C, SMC2, SMC3, SMC4, EML4, CCNB1, MAD2L1, CCNB2, NOLC1, SPAG5, ZWINT, BUB1B, MAPRE1* |
| GOTERM_BP_FAT | GO:0022403~  cell cycle phase | Upregulation | 1.04E-11 | 56 | *KIF23, PRC1, DBF4, TTK, AURKA, GTSE1, FAM83D, KIF2C, RAD21, CCNA2, ASPM, CDK1, CDC6, KIF11, ANAPC5, TPX2, NUSAP1, PBK, UBE2C, PPP1CB, EML4, INHBA, MAD2L1, SPAG5, ZWINT, BUB1B, MAPRE1, KPNA2, NEK2, ANLN, CEP55, ITGB1, CCNG2, BUB1, ZWILCH, BUB3, HELLS, TRIP13, MSH6, MKI67, CKAP5, DLGAP5, NUF2, CENPF, BIRC5, NDC80, CDC20, CDKN3, SMC2, SMC3, SMC4, CCNB1, CCNB2, NOLC1, GSPT1, CKS2* |
| GOTERM_BP_FAT | GO:0000279~  M phase | Upregulation | 1.23E-11 | 49 | *KIF23, PRC1, TTK, AURKA, FAM83D, KIF2C, RAD21, CCNA2, ASPM, CDC6, CDK1, KIF11, ANAPC5, TPX2, NUSAP1, PBK, UBE2C, EML4, MAD2L1, SPAG5, ZWINT, BUB1B, MAPRE1, KPNA2, NEK2, ANLN, CEP55, CCNG2, BUB1, ZWILCH, HELLS, BUB3, TRIP13, MSH6, MKI67, CKAP5, DLGAP5, NUF2, CENPF, NDC80, BIRC5, CDC20, SMC2, SMC3, SMC4, CCNB1, CCNB2, NOLC1, CKS2* |
| GOTERM_BP_FAT | GO:0007049~  cell cycle | Upregulation | 1.33E-11 | 81 | *KIF23, PRC1, DBF4, E2F8, TTK, AURKA, WTAP, GTSE1, CCNE2, FAM83D, KIF2C, RAD21, CASP8AP2, FANCI, CCNA2, ASPM, CDK1, CDC6, RBBP4, KIF11, ANAPC5, TPX2, NUSAP1, PBK, CDC5L, UBE2C, PPP1CB, EML4, MAPK1, INHBA, UHRF1, MAD2L1, SPAG5, ZWINT, BUB1B, MAPRE1, SIAH2, KPNA2, NEK2, FOXM1, ANLN, CEP55, CCNG2, ITGB1, UHMK1, PSMB4, MACF1, CENPA, RB1CC1, BUB1, THBS1, ZWILCH, HELLS, BUB3, TRIP13, CKAP2, MSH6, PARD6B, MKI67, MSH2, CKAP5, DLGAP5, NUF2, CENPF, CDC20, BIRC5, NDC80, RACGAP1, CDKN3, SMC2, SMC3, SMC4, CCNB1, CCNB2, GSPT1, PSMD12, NOLC1, IRF6, PSMD11, PTP4A1, CKS2* |
| GOTERM_BP_FAT | GO:0022402~  cell cycle process | Upregulation | 2.59E-11 | 66 | *KIF23, PRC1, DBF4, TTK, AURKA, GTSE1, FAM83D, KIF2C, RAD21, CCNA2, ASPM, CDK1, CDC6, KIF11, ANAPC5, TPX2, NUSAP1, PBK, UBE2C, PPP1CB, EML4, INHBA, MAD2L1, SPAG5, ZWINT, BUB1B, MAPRE1, KPNA2, NEK2, ANLN, CEP55, ITGB1, CCNG2, UHMK1, PSMB4, MACF1, CENPA, BUB1, ZWILCH, THBS1, BUB3, HELLS, TRIP13, MSH6, MKI67, MSH2, CKAP5, DLGAP5, NUF2, CENPF, CDC20, BIRC5, NDC80, RACGAP1, CDKN3, SMC2, SMC3, SMC4, CCNB1, CCNB2, GSPT1, PSMD12, NOLC1, IRF6, PSMD11, CKS2* |
| GOTERM_BP_FAT | GO:0051301~  cell division | Upregulation | 8.27E-10 | 43 | *KIF23, PRC1, NEK2, ANLN, CEP55, CCNG2, FAM83D, CCNE2, CDC42, RAD21, BUB1, ZWILCH, CCNA2, RASA1, HELLS, ASPM, PARD6B, CDK1, CDC6, KIF11, ANAPC5, CKAP5, NUF2, CENPF, NUSAP1, CDC20, BIRC5, NDC80, RACGAP1, UBE2C, SMC2, PPP1CB, SMC3, SMC4, CCNB1, MAD2L1, CCNB2, SPAG5, ZWINT, CFL1, CKS2, BUB1B, MAPRE1* |
| GOTERM_BP_FAT | GO:0007059~  chromosome segregation | Upregulation | 8.48E-06 | 18 | *NEK2, DLGAP5, NUF2, CENPF, NUSAP1, NDC80, BIRC5, SMC2, SMC3, SMC4, RAD21, MAD2L1, PSEN1, RIOK3, ZWINT, ARL8B, TOP2A, BUB3* |
| GOTERM_BP_FAT | GO:0000075~  cell cycle checkpoint | Upregulation | 8.07E-06 | 19 | *CDC6, CDK1, BRCC3, MSH2, CENPF, TTK, BIRC5, TRRAP, CCNG2, GTSE1, CCNE2, MAD2L1, ZWINT, BUB1, BUB1B, ZWILCH, CCNA2, BUB3, DLG1* |
| GOTERM_BP_FAT | GO:0007093~  mitotic cell cycle checkpoint | Upregulation | 2.74E-05 | 13 | *CDK1, TTK, CENPF, TRRAP, GTSE1, MAD2L1, ZWINT, BUB1, BUB1B, ZWILCH, CCNA2, BUB3, DLG1* |
| GOTERM_BP_FAT | GO:0006323~  DNA packaging | Upregulation | 7.61E-05 | 20 | *HIST2H2AA3, HIST1H2BD, HIST1H2BF, HIST1H2BG, HIST1H2BH, NUSAP1, HAT1, SMC2, SMC4, H2BFS, SET, HIST1H2BK, H2AFV, CENPA, HIST2H2BE, HIST1H2BI, H2AFZ, H2AFY, HIST1H3D, ASF1A, TOP2A, HELLS* |
| GOTERM_BP_FAT | GO:0051726~  regulation of cell cycle | Upregulation | 8.47E-05 | 36 | *NEK2, TTK, ANLN, TRRAP, ITGB1, CCNG2, GTSE1, CITED2, CCNE2, CDC42, BUB1, RHOB, ZWILCH, CCNA2, BUB3, HSPA8, DLG1, CDK1, CDC6, PTPRC, BRCC3, MSH2, DLGAP5, CENPF, NUSAP1, BIRC5, CDKN3, UBE2C, PPP1CB, CCNB1, INHBA, MAD2L1, ZWINT, CKS2, BUB1B, GADD45B* |
| GOTERM_BP_FAT | GO:0000226~  microtubule cytoskeleton organization | Upregulation | 1.47E-04 | 22 | *KIF23, KIF11, PRC1, CKAP5, NEK2, TTK, NUSAP1, AURKA, NDC80, ARPC4, UBE2C, UBE2B, SMC3, SS18, KIF2C, RANBP9, MACF1, SPAG5, CENPA, ZWINT, CKS2, BUB1B* |
| GOTERM_BP_FAT | GO:0042981~  regulation of apoptosis | Upregulation | 1.65E-04 | 64 | *MMP9, EIF5A, PMAIP1, FOXO3, CITED2, CD44, ATG5, CASP8AP2, TIA1, RHOA, PIK3CA, PSENEN, FOXO3B, TOP2A, NET1, CDK1, BCL2A1, PRKCI, MBD4, ECT2, BCL2L11, MAPK1, INHBA, PSEN1, TNFSF13B, IGF2R, CFL1, VEGFA, CTSB, TNFAIP3, BCLAF1, YWHAZ, CCL2, MCL1, ERBB2, SOX4, PRKDC, CDH1, HSPA1B, GCH1, SERINC3, COMP, RB1CC1, RAC1, DYRK2, CD24, THBS1, HELLS, RASA1, MSH6, PTPRC, CFLAR, VAV3, MSH2, CREB1, BIRC5, STAT1, YWHAE, PPIF, NRAS, HSP90B1, GSPT1, HSPD1, PERP, IFI6* |
| GOTERM_BP_FAT | GO:0043067~  regulation of programmed cell death | Upregulation | 2.16E-04 | 64 | *MMP9, EIF5A, PMAIP1, FOXO3, CITED2, CD44, ATG5, CASP8AP2, TIA1, RHOA, PIK3CA, PSENEN, FOXO3B, TOP2A, NET1, CDK1, BCL2A1, PRKCI, MBD4, ECT2, BCL2L11, MAPK1, INHBA, PSEN1, TNFSF13B, IGF2R, CFL1, VEGFA, CTSB, TNFAIP3, BCLAF1, YWHAZ, CCL2, MCL1, ERBB2, SOX4, PRKDC, CDH1, HSPA1B, GCH1, SERINC3, COMP, RB1CC1, RAC1, DYRK2, CD24, THBS1, HELLS, RASA1, MSH6, PTPRC, CFLAR, VAV3, MSH2, CREB1, BIRC5, STAT1, YWHAE, PPIF, NRAS, HSP90B1, GSPT1, HSPD1, PERP, IFI6* |
| GOTERM_BP_FAT | GO:0010941~  regulation of cell death | Upregulation | 2.27E-04 | 64 | *MMP9, EIF5A, PMAIP1, FOXO3, CITED2, CD44, ATG5, CASP8AP2, TIA1, RHOA, PIK3CA, PSENEN, FOXO3B, TOP2A, NET1, CDK1, BCL2A1, PRKCI, MBD4, ECT2, BCL2L11, MAPK1, INHBA, PSEN1, TNFSF13B, IGF2R, CFL1, VEGFA, CTSB, TNFAIP3, BCLAF1, YWHAZ, CCL2, MCL1, ERBB2, SOX4, PRKDC, CDH1, HSPA1B, GCH1, SERINC3, COMP, RB1CC1, RAC1, DYRK2, CD24, THBS1, HELLS, RASA1, MSH6, PTPRC, CFLAR, VAV3, MSH2, CREB1, BIRC5, STAT1, YWHAE, PPIF, NRAS, HSP90B1, GSPT1, HSPD1, PERP, IFI6* |
| GOTERM_BP_FAT | GO:0007051~  spindle organization | Upregulation | 2.51E-04 | 12 | *KIF23, KIF11, PRC1, SPAG5, ZWINT, CKS2, BUB1B, TTK, NDC80, AURKA, UBE2C, SMC3* |
| GOTERM_BP_FAT | GO:0007017~  microtubule-based process | Upregulation | 3.55E-04 | 29 | *KIF14, KIF23, KIF4A, KIF11, PRC1, CKAP5, NEK2, TTK, NUSAP1, AURKA, NDC80, ARPC4, UBE2C, UBE2B, SMC3, GTSE1, EML4, KIF2C, SS18, RANBP9, MACF1, SPAG5, CENPA, ZWINT, CKS2, BUB1B, KPNA2, KIF2A, KIF20A* |
| GOTERM_BP_FAT | GO:0065004~  protein-DNA complex assembly | Upregulation | 6.61E-04 | 16 | *HIST2H2AA3, HIST1H2BD, HIST1H2BF, HIST1H2BG, HIST1H2BH, CENPF, SET, H2BFS, HIST1H2BK, H2AFV, HIST2H2BE, CENPA, HIST1H2BI, H2AFZ, H2AFY, HIST1H3D, ASF1A, HELLS* |
| GOTERM_BP_FAT | GO:0007346~  regulation of mitotic cell cycle | Upregulation | 6.68E-04 | 21 | *CDC6, CDK1, NEK2, DLGAP5, CENPF, TTK, NUSAP1, ANLN, BIRC5, TRRAP, UBE2C, GTSE1, CDC42, MAD2L1, ZWINT, BUB1, BUB1B, ZWILCH, CCNA2, BUB3, DLG1* |
| GOTERM_BP_FAT | GO:0044265~  cellular macromolecule catabolic process | Upregulation | 7.58E-04 | 57 | *NCBP1, UBE2G1, PPP2R5C, SAE1, ISG15, FBXO28, DDA1, PSENEN, RANBP2, CDK1, TBL1XR1, BRCC3, ANAPC5, DTL, MAGOH, ENC1, UBE2J1, MBD4, UBE2H, UBE2C, UBE2B, UBE2N, UHRF1, DCUN1D1, MAD2L1, PSEN1, UBE2M, BUB1B, UBD, SIAH2, TNFAIP3, UBE2S, XRN2, UBE2T, DERL1, UBE2V1, UBA6, HSPA1B, OAS2, EDEM3, PSMB4, ZFP36L2, SUMO2, CACYBP, USP38, USP34, BUB3, FBXO45, FEN1, SMG1, CDC20, WSB1, CCNB1, HSP90B1, GSPT1, PSMD12, PSMD11* |
| GOTERM_BP_FAT | GO:0034621~  cellular macromolecular complex subunit organization | Upregulation | 7.65E-04 | 35 | *NCBP1, HIST2H2AA3, FKBP4, DICER1, FKBP1A, ARPC4, ANLN, KIF2C, H2BFS, SET, PICALM, PAK2, H2AFV, HIST1H2BK, CENPA, HIST1H2BI, RAC1, H2AFZ, H2AFY, HSPA4, ASF1A, HELLS, SRPK2, HIST1H2BD, TSR1, HIST1H2BF, HIST1H2BG, HIST1H2BH, SMAD4, CENPF, TMEM70, MBNL1, HIST2H2BE, IPO5, HIST1H3D, HSPD1, XRN2* |
| GOTERM_BP_FAT | GO:0007242~  intracellular signaling cascade | Upregulation | 9.97E-04 | 85 | *STK38, AURKA, RAB1A, GTSE1, CDC42, CXCR4, RHOA, ANP32A, PIK3CA, RHOB, CCNA2, TOP2A, NET1, SRPK2, CDK1, ARL1, MYO6, BRCC3, PIK3C2A, RAB4A, G3BP2, PRKCI, MBD4, UBE2C, ECT2, NRIP1, MAPK1, SS18, MAP4K5, RAB18, PSEN1, NCOA3, SPAG5, GNB1, MED17, ZWINT, KRIT1, CFL1, COL1A2, RAB5A, RAB14, BUB1B, ARL8B, SIAH2, GADD45B, RAB10, MED1, CCL2, ERBB2, MAPKAPK2, ARFGEF2, TYMS, RB1CC1, RAC1, DYRK2, THBS1, FEN1, RASA1, ARHGDIB, MSH6, PTPRC, GDI2, VAV3, MSH2, NDC80, RACGAP1, STAT1, YWHAE, WSB1, NRAS, RAB31, YWHAH, RGS1, ARF1, PDE7A, CNIH4, PRKAR1A, THRAP3, ARF4, PCNA, CKS2, JAK1, RHEB, RIT1, CLEC7A* |
| GOTERM_BP_FAT | GO:0034622~  cellular macromolecular complex assembly | Upregulation | 0.0011303 | 32 | *NCBP1, HIST2H2AA3, FKBP4, DICER1, FKBP1A, ARPC4, ANLN, H2BFS, SET, PICALM, H2AFV, HIST1H2BK, CENPA, HIST1H2BI, RAC1, H2AFZ, H2AFY, HSPA4, ASF1A, HELLS, SRPK2, HIST1H2BD, TSR1, HIST1H2BF, HIST1H2BG, HIST1H2BH, SMAD4, CENPF, TMEM70, MBNL1, HIST2H2BE, IPO5, HIST1H3D, HSPD1* |
| GOTERM_BP_FAT | GO:0015031~  protein transport | Upregulation | 0.0013394 | 58 | *CHMP4B, TIMM17A, EIF5A, CANX, RAB1A, CXCL10, SSR1, RHOB, RANBP2, KDELR1, KDELR3, KDELR2, MYO6, RAB4A, PRKCI, MAPK1, PSEN1, TNFSF13B, RAB18, VAMP7, IPO5, RAB5A, RAB14, KPNA4, COL1A1, SRP72, RAB10, KPNA2, KPNA1, LCP2, YWHAZ, DERL1, COPZ1, SNX2, SNX4, SFT2D1, MIA3, TMED2, MACF1, SNAP23, CD24, TRAM1, SEC61A1, GDI2, VTA1, CENPF, YWHAE, RAB31, HSP90B1, YWHAH, ARF1, CHML, TRPS1, ARF4, PCNA, YIPF5, KIF20A, TOB1* |
| GOTERM_BP_FAT | GO:0031497~  chromatin assembly | Upregulation | 0.0013586 | 15 | *HIST2H2AA3, HIST1H2BD, HIST1H2BF, HIST1H2BG, HIST1H2BH, SET, H2BFS, HIST1H2BK, H2AFV, HIST2H2BE, CENPA, HIST1H2BI, H2AFZ, H2AFY, HIST1H3D, ASF1A, HELLS* |
| GOTERM_BP_FAT | GO:0007010~  cytoskeleton organization | Upregulation | 0.001426 | 39 | *KIF23, CCL3, PRC1, CNN3, NEK2, CALD1, TTK, ANLN, ARPC4, AURKA, ARPC5, ITGB1, CDC42, KIF2C, RANBP9, EZR, MACF1, PAK2, CENPA, RAC1, RHOA, DLG1, ARHGDIB, KIF11, CKAP5, PRKCI, NUSAP1, NDC80, RACGAP1, UBE2C, UBE2B, SMC3, NRAS, SS18, SPAG5, ZWINT, CFL1, CKS2, BUB1B* |
| GOTERM_BP_FAT | GO:0048015~  phosphoinositide-mediated signaling | Upregulation | 0.0014509 | 15 | *PIK3C2A, ERBB2, AURKA, NDC80, UBE2C, TYMS, GNB1, SPAG5, ZWINT, PCNA, CKS2, BUB1B, PIK3CA, TOP2A, FEN1* |
| GOTERM_BP_FAT | GO:0045184~  establishment of protein localization | Upregulation | 0.001555 | 58 | *CHMP4B, TIMM17A, EIF5A, CANX, RAB1A, CXCL10, SSR1, RHOB, RANBP2, KDELR1, KDELR3, KDELR2, MYO6, RAB4A, PRKCI, MAPK1, PSEN1, TNFSF13B, RAB18, VAMP7, IPO5, RAB5A, RAB14, KPNA4, COL1A1, SRP72, RAB10, KPNA2, KPNA1, LCP2, YWHAZ, DERL1, COPZ1, SNX2, SNX4, SFT2D1, MIA3, TMED2, MACF1, SNAP23, CD24, TRAM1, SEC61A1, GDI2, VTA1, CENPF, YWHAE, RAB31, HSP90B1, YWHAH, ARF1, CHML, TRPS1, ARF4, PCNA, YIPF5, KIF20A, TOB1* |
| GOTERM_BP_FAT | GO:0010498~  proteasomal protein catabolic process | Upregulation | 0.0018048 | 16 | *TBL1XR1, CDK1, DERL1, ANAPC5, PPP2R5C, CDC20, EDEM3, UBE2C, CCNB1, PSMB4, HSP90B1, MAD2L1, PSMD12, PSMD11, BUB1B, BUB3* |
| GOTERM_BP_FAT | GO:0043161~  proteasomal ubiquitin-dependent protein catabolic process | Upregulation | 0.0018048 | 16 | *TBL1XR1, CDK1, DERL1, ANAPC5, PPP2R5C, CDC20, EDEM3, UBE2C, CCNB1, PSMB4, HSP90B1, MAD2L1, PSMD12, PSMD11, BUB1B, BUB3* |
| GOTERM_BP_FAT | GO:0006955~  immune response | Upregulation | 0.0022385 | 53 | *KYNU, IGHG3, S100A7, IFI44L, CXCL11, IGHM, PNP, CXCL10, NUDCD1, CXCR4, CD46, FCGR3B, CNPY3, CTSS, CD164, HLA-DQA1, CD86, PSEN1, TNFSF13B, VAMP7, VEGFA, CTSC, HLA-DPA1, LCP2, HLA-DRA, HLA-DQB1, YWHAZ, CCL3, CCL2, OAS3, CXCL9, RSAD2, PRKDC, OAS1, OAS2, C1S, GCH1, FCGR1A, FCGR1B, IGHA1, FCER1G, CD24, THBS1, IGKC, ARHGDIB, MSH6, PTPRC, MSH2, DDX58, RGS1, ILF2, HSPD1, CLEC7A, IFI6* |
| GOTERM_BP_FAT | GO:0009057~  macromolecule catabolic process | Upregulation | 0.0021984 | 58 | *NCBP1, UBE2G1, PPP2R5C, SAE1, ISG15, FBXO28, DDA1, PSENEN, RANBP2, CDK1, TBL1XR1, BRCC3, ANAPC5, DTL, MAGOH, ENC1, UBE2J1, MBD4, UBE2H, UBE2C, UBE2B, UBE2N, UHRF1, DCUN1D1, MAD2L1, PSEN1, UBE2M, BUB1B, UBD, SIAH2, TNFAIP3, UBE2S, XRN2, UBE2T, DERL1, UBE2V1, UBA6, FKBP1A, HSPA1B, OAS2, EDEM3, PSMB4, ZFP36L2, SUMO2, CACYBP, USP38, USP34, BUB3, FBXO45, FEN1, SMG1, CDC20, WSB1, CCNB1, HSP90B1, GSPT1, PSMD12, PSMD11* |
| GOTERM_BP_FAT | GO:0051276~  chromosome organization | Upregulation | 0.0025414 | 41 | *HIST2H2AA3, NEK2, MORF4L2, EZH2, PRKDC, HAT1, TRRAP, H2BFS, SET, H2AFV, HIST1H2BK, CENPA, HIST1H2BI, H2AFZ, H2AFY, BCOR, ASF1A, TOP2A, HELLS, BUB3, MSH6, TBL1XR1, RBBP4, HIST1H2BD, BRCC3, HIST1H2BF, MSH2, HIST1H2BG, DLGAP5, HIST1H2BH, CENPF, NUSAP1, NDC80, SMC2, UBE2B, SMC3, SMC4, UBE2N, MAD2L1, HIST2H2BE, ZWINT, SMARCC1, HIST1H3D* |
| GOTERM_BP_FAT | GO:0008104~  protein localization | Upregulation | 0.0028035 | 63 | *CHMP4B, TIMM17A, EIF5A, CANX, RAB1A, CXCL10, SSR1, CDC42, RHOB, RANBP2, KDELR1, KDELR3, KDELR2, MYO6, RAB4A, PRKCI, G3BP2, MAPK1, PSEN1, TNFSF13B, RAB18, VAMP7, IPO5, RAB5A, RAB14, SRP72, KPNA4, COL1A1, RAB10, KPNA2, KPNA1, LCP2, YWHAZ, DERL1, COPZ1, SNX2, SNX4, SFT2D1, MIA3, EZR, TMED2, MACF1, SNAP23, CD24, TRAM1, SEC61A1, SRGN, GDI2, VTA1, CENPF, NDC80, YWHAE, RAB31, HSP90B1, YWHAH, ARF1, CHML, TRPS1, ARF4, PCNA, YIPF5, KIF20A, TOB1* |
| GOTERM_BP_FAT | GO:0065003~  macromolecular complex assembly | Upregulation | 0.0029934 | 51 | *HIST2H2AA3, NCBP1, DICER1, RANBP9, PICALM, H2BFS, HIST1H2BK, PAK2, H2AFV, HIST1H2BI, H2AFZ, H2AFY, SRPK2, TMEM70, MBNL1, HIST2H2BE, MED17, RRM2, IPO5, RRM1, VAMP3, MED1, POLR2K, FKBP4, POLR2J, ARPC4, CDH1, FKBP1A, ANLN, HPRT1, GCH1, SET, CENPA, ALOX5AP, RAC1, HSPA4, SKIL, ASF1A, HELLS, SOAT1, PARD6B, HIST1H2BD, TSR1, HIST1H2BF, HIST1H2BG, HIST1H2BH, SMAD4, TRIM27, CENPF, EPRS, THRAP3, HIST1H3D, HSPD1* |
| GOTERM_BP_FAT | GO:0006334~  nucleosome assembly | Upregulation | 0.0030926 | 14 | *HIST2H2AA3, HIST1H2BD, HIST1H2BF, HIST1H2BG, HIST1H2BH, SET, H2BFS, HIST1H2BK, H2AFV, HIST2H2BE, CENPA, HIST1H2BI, H2AFZ, H2AFY, HIST1H3D, ASF1A* |
| GOTERM_BP_FAT | GO:0051603~  proteolysis involved in cellular protein catabolic process | Upregulation | 0.0036562 | 47 | *DERL1, UBE2G1, PPP2R5C, UBE2V1, UBA6, SAE1, EDEM3, PSMB4, SUMO2, ISG15, CACYBP, FBXO28, USP38, DDA1, PSENEN, RANBP2, USP34, FBXO45, BUB3, TBL1XR1, CDK1, BRCC3, ANAPC5, DTL, ENC1, UBE2J1, CDC20, UBE2H, UBE2C, UBE2B, UBE2N, WSB1, CCNB1, UHRF1, HSP90B1, DCUN1D1, MAD2L1, PSMD12, PSEN1, PSMD11, UBE2M, BUB1B, UBD, SIAH2, TNFAIP3, UBE2S, UBE2T* |
| GOTERM_BP_FAT | GO:0043933~  macromolecular complex subunit organization | Upregulation | 0.0039237 | 53 | *HIST2H2AA3, NCBP1, DICER1, KIF2C, RANBP9, PICALM, H2BFS, HIST1H2BK, PAK2, H2AFV, HIST1H2BI, H2AFZ, H2AFY, SRPK2, TMEM70, MBNL1, HIST2H2BE, MED17, RRM2, IPO5, RRM1, VAMP3, XRN2, MED1, POLR2K, FKBP4, POLR2J, ARPC4, CDH1, FKBP1A, ANLN, HPRT1, GCH1, SET, CENPA, ALOX5AP, RAC1, HSPA4, SKIL, ASF1A, HELLS, SOAT1, PARD6B, HIST1H2BD, TSR1, HIST1H2BF, HIST1H2BG, HIST1H2BH, SMAD4, TRIM27, CENPF, EPRS, THRAP3, HIST1H3D, HSPD1* |
| GOTERM_BP_FAT | GO:0044257~  cellular protein catabolic process | Upregulation | 0.003959 | 47 | *DERL1, UBE2G1, PPP2R5C, UBE2V1, UBA6, SAE1, EDEM3, PSMB4, SUMO2, ISG15, CACYBP, FBXO28, USP38, DDA1, PSENEN, RANBP2, USP34, FBXO45, BUB3, TBL1XR1, CDK1, BRCC3, ANAPC5, DTL, ENC1, UBE2J1, CDC20, UBE2H, UBE2C, UBE2B, UBE2N, WSB1, CCNB1, UHRF1, HSP90B1, DCUN1D1, MAD2L1, PSMD12, PSEN1, PSMD11, UBE2M, BUB1B, UBD, SIAH2, TNFAIP3, UBE2S, UBE2T* |
| GOTERM_BP_FAT | GO:0019941~  modification-dependent protein catabolic process | Upregulation | 0.0049457 | 45 | *DERL1, UBE2G1, PPP2R5C, UBE2V1, UBA6, SAE1, EDEM3, PSMB4, SUMO2, ISG15, CACYBP, FBXO28, USP38, DDA1, RANBP2, USP34, FBXO45, BUB3, CDK1, TBL1XR1, BRCC3, ANAPC5, DTL, ENC1, UBE2J1, CDC20, UBE2H, UBE2C, UBE2B, UBE2N, CCNB1, WSB1, UHRF1, HSP90B1, DCUN1D1, MAD2L1, PSMD12, PSMD11, UBE2M, BUB1B, UBD, SIAH2, TNFAIP3, UBE2S, UBE2T* |
| GOTERM_BP_FAT | GO:0043632~  modification-dependent macromolecule catabolic process | Upregulation | 0.0049457 | 45 | *DERL1, UBE2G1, PPP2R5C, UBE2V1, UBA6, SAE1, EDEM3, PSMB4, SUMO2, ISG15, CACYBP, FBXO28, USP38, DDA1, RANBP2, USP34, FBXO45, BUB3, CDK1, TBL1XR1, BRCC3, ANAPC5, DTL, ENC1, UBE2J1, CDC20, UBE2H, UBE2C, UBE2B, UBE2N, CCNB1, WSB1, UHRF1, HSP90B1, DCUN1D1, MAD2L1, PSMD12, PSMD11, UBE2M, BUB1B, UBD, SIAH2, TNFAIP3, UBE2S, UBE2T* |
| GOTERM_BP_FAT | GO:0008283~  cell proliferation | Upregulation | 0.0049766 | 37 | *CDV3, OSMR, ERBB2, E2F8, FKBP1A, HPRT1, KIF2C, ZFP36L2, CXCR4, RAC1, BUB1, COL8A1, HELLS, BUB3, ASPM, DLG1, GINS1, PTPRC, MKI67, DLGAP5, TRIM27, TPX2, CENPF, RACGAP1, UHRF1, CD86, GLUL, TNFSF13B, PSEN1, GNB1, IRF6, VEGFA, CKS2, PCNA, BUB1B, HSPD1, MAPRE1* |
| GOTERM_BP_FAT | GO:0016192~  vesicle-mediated transport | Upregulation | 0.0051309 | 45 | *YWHAZ, CCL3, GNAI3, COPZ1, SNX2, SNX4, ARFGEF2, ARFGEF1, RAB1A, MIA3, PICALM, TMED2, FOLR1, FCGR1A, RAC1, RHOB, FCER1G, SNAP23, CD24, THBS1, KDELR1, HSPA8, KDELR3, ARL1, KDELR2, MYO6, VAV3, NCALD, PRKCI, M6PR, NRAS, RAB18, TFRC, PSEN1, ARF1, IGF2R, VAMP7, ARF4, RAB14, RAB5A, VAMP3, CPNE3, YIPF5, CLEC7A, KIF20A* |
| GOTERM_BP_FAT | GO:0010033~  response to organic substance | Upregulation | 0.0050297 | 53 | *HSP90AB1, KYNU, OSMR, DICER1, AURKA, PMAIP1, ASAH1, ACTR3, HSPH1, CD44, DNAJC3, CCNA2, PRKCI, UBE2B, MAPK1, GLUL, SDC1, GNB1, SQLE, CFL1, CTSC, NFE2L2, COL1A1, DERL1, CCL2, MCL1, ERBB2, COL3A1, PRKDC, CDH1, FKBP1A, HSPA1B, C1S, EDEM3, HPRT1, TTC3, GCH1, HSPA4, CD24, THBS1, HSPA8, SPP1, MAT2A, MSH2, CREB1, GGH, STAT1, TFRC, PRKAR1A, DNAJB1, HSPD1, CLEC7A, UBXN4* |
| GOTERM_BP_FAT | GO:0007264~  small GTPase mediated signal transduction | Upregulation | 0.0049255 | 29 | *MAPKAPK2, RAB1A, CDC42, RAC1, RHOA, RHOB, ARHGDIB, ARL1, GDI2, VAV3, RAB4A, G3BP2, NRAS, MAPK1, RAB31, RAB18, ARF1, GNB1, KRIT1, CFL1, ARF4, COL1A2, RAB5A, RAB14, RHEB, RIT1, ARL8B, SIAH2, RAB10* |
| GOTERM_BP_FAT | GO:0000070~  mitotic sister chromatid segregation | Upregulation | 0.0055843 | 9 | *MAD2L1, NEK2, ZWINT, DLGAP5, NUSAP1, NDC80, SMC2, BUB3, SMC4* |
| GOTERM_BP_FAT | GO:0006928~  cell motion | Upregulation | 0.0055207 | 39 | *CTHRC1, CCL3, CCL2, HMGCR, CALD1, ERBB2, PRKDC, FPR3, ITGB2, ARPC5, ITGB1, TPM4, TPM3, ACTR3, CD9, ACTR2, MACF1, CD44, CXCR4, PEX2, RAC1, CD24, B3GNT2, THBS1, ARHGDIB, FN1, ACTB, SGPL1, S100P, VAV3, MSH2, YWHAE, COL5A1, PLAUR, PARP9, PSEN1, CFL1, VCAN, PLAU* |
| GOTERM_BP_FAT | GO:0000819~  sister chromatid segregation | Upregulation | 0.0065832 | 9 | *MAD2L1, NEK2, ZWINT, DLGAP5, NUSAP1, NDC80, SMC2, BUB3, SMC4* |
| GOTERM_BP_FAT | GO:0030163~  protein catabolic process | Upregulation | 0.0067434 | 47 | *DERL1, UBE2G1, PPP2R5C, UBE2V1, UBA6, SAE1, EDEM3, PSMB4, SUMO2, ISG15, CACYBP, FBXO28, USP38, DDA1, PSENEN, RANBP2, USP34, FBXO45, BUB3, TBL1XR1, CDK1, BRCC3, ANAPC5, DTL, ENC1, UBE2J1, CDC20, UBE2H, UBE2C, UBE2B, UBE2N, WSB1, CCNB1, UHRF1, HSP90B1, DCUN1D1, MAD2L1, PSMD12, PSEN1, PSMD11, UBE2M, BUB1B, UBD, SIAH2, TNFAIP3, UBE2S, UBE2T* |
| GOTERM_BP_FAT | GO:0034728~  nucleosome organization | Upregulation | 0.0067639 | 14 | *HIST2H2AA3, HIST1H2BD, HIST1H2BF, HIST1H2BG, HIST1H2BH, SET, H2BFS, HIST1H2BK, H2AFV, HIST2H2BE, CENPA, HIST1H2BI, H2AFZ, H2AFY, HIST1H3D, ASF1A* |
| GOTERM_BP_FAT | GO:0012501~  programmed cell death | Upregulation | 0.0084709 | 46 | *MCL1, PRKDC, ITGB2, PMAIP1, FOXO3, GREM1, RAD21, CASP8AP2, ATG5, PAK2, CXCR4, TIA1, BAG2, RAC1, RHOB, PSENEN, DYRK2, CD24, FOXO3B, THBS1, TOP2A, SRGN, PHLDA2, NET1, CKAP2, CFLAR, SGPL1, VAV3, MSH2, BCL2A1, BIRC5, STAT1, YWHAE, ECT2, BCL2L11, VDAC1, NRAS, GSPT1, PSEN1, SULF1, BUB1B, HSPD1, SIAH2, TNFAIP3, PERP, GADD45B, IFI6* |
| GOTERM_BP_FAT | GO:0006986~  response to unfolded protein | Upregulation | 0.0084252 | 12 | *HSP90AB1, HSPH1, DERL1, HSPA4, EDEM3, NFE2L2, DNAJB1, HSPD1, HSPA1B, DNAJC3, UBXN4, HSPA8* |
| GOTERM_BP_FAT | GO:0006259~  DNA metabolic process | Upregulation | 0.0087844 | 40 | *DBF4, MORF4L2, PRKDC, TK1, CCNE2, NONO, TYMS, SET, RAD21, POLE3, FANCI, ASF1A, TOP2A, HELLS, FEN1, TRIP13, GINS1, MSH6, CDC6, RBBP4, BRCC3, RAD51AP1, DTL, MSH2, CENPF, SMG1, MBD4, MCM4, UBE2B, SMC3, UBE2N, RFC5, UHRF1, RRM2, RRM1, PCNA, HSPD1, KPNA2, XRN2, MED1* |
| GOTERM_BP_FAT | GO:0022610~  biological adhesion | Downregulation | 0.0138972 | 19 | *CLDN8, TNXB, TNXA, CLDN5, PTPRS, CX3CL1, CLDN11, JUP, VWF, LAMB3, CD36, SRPX, SORBS1, FCGBP, JAM2, DST, BOC, PARVA, AOC3, DPT* |
| GOTERM_BP_FAT | GO:0007155~  cell adhesion | Downregulation | 0.0203897 | 19 | *CLDN8, TNXB, TNXA, CLDN5, PTPRS, CX3CL1, CLDN11, JUP, VWF, LAMB3, CD36, SRPX, SORBS1, FCGBP, JAM2, DST, BOC, PARVA, AOC3, DPT* |
| GOTERM_BP_FAT | GO:0051259~  protein oligomerization | Downregulation | 0.0262349 | 10 | *TRIM4, JUP, VWF, CAV1, CRYAB, TP63, ADIPOQ, AKR1C1, PARVA, ANGPTL4* |
| GOTERM_BP_FAT | GO:0007010~  cytoskeleton organization | Downregulation | 0.03309 | 14 | *CAV1, TNXB, TNXA, SORBS1, CRYAB, DMD, ABLIM3, KRT14, MYH11, OPHN1, SYNM, CNN1, DST, EHD2, PARVA* |
| GOTERM_BP_FAT | GO:0051260~  protein homooligomerization | Downregulation | 0.0451428 | 7 | *VWF, CAV1, CRYAB, TP63, ADIPOQ, AKR1C1, ANGPTL4* |
