# Supplementary material for: An in-silico method leads to recognition of hub genes and crucial pathways in survival of patients with breast cancer
Source: Sci Rep. 2020 Oct 30;10:18770. doi: 10.1038/s41598-020-76024-2 (PMC7603345; doi:10.1038/s41598-020-76024-2)
Supplement: Supplementary file 6 — Supplementary Information 6. [file 41598_2020_76024_MOESM6_ESM.docx]

An in-silico method leads to recognition of hub genes and crucial pathways in survival of patients with breast cancer

Sepideh Dashti^1^, Mohammad Taheri^2^, Soudeh Ghafouri-Fard^1^*

1. Department of Medical Genetics, Shahid Beheshti University of Medical Sciences, Tehran, Iran
2. Urogenital Stem Cell Research Center, Shahid Beheshti University of Medical Sciences, Tehran, Iran

Table S5. The result of GO enrichment analysis (GO for DEGs, Cellular component).

| **Category** | **Term** | **SampleGroup** | **Qvalue** | **Count** | **Genes** |
| --- | --- | --- | --- | --- | --- |
| GOTERM_CC_FAT | GO:0005819~  spindle | Upregulation | 2.51E-09 | 29 | *KIF23, KIF4A, PRC1, NEK2, TTK, AURKA, FAM83D, BUB1, ASPM, CKAP2, KIF14, CDC6, CDK1, TBL1XR1, KIF11, DLGAP5, TPX2, CENPF, NUSAP1, BIRC5, CDC20, RACGAP1, SMC3, MAD2L1, SPAG5, PTP4A1, BUB1B, ARL8B, MAPRE1* |
| GOTERM_CC_FAT | GO:0005694~  chromosome | Upregulation | 9.48E-07 | 48 | *HIST2H2AA3, PRPF4B, NEK2, CITED2, KIF2C, SUMO2, H2BFS, CSNK2A1, RAD21, HIST1H2BK, H2AFV, CENPA, FANCI, HIST1H2BI, BUB1, H2AFZ, H2AFY, ZWILCH, TOP2A, BUB3, HELLS, MSH6, RBBP4, HIST1H2BD, MKI67, HIST1H2BF, CREB1, HIST1H2BG, HIST1H2BH, NUF2, CENPF, BIRC5, NDC80, MBD4, CENPK, UBE2B, SMC2, SMC3, SMC4, RFC5, MAD2L1, BAZ1A, PSEN1, HIST2H2BE, SPAG5, ZWINT, SMARCC1, PCNA, BUB1B, HIST1H3D* |
| GOTERM_CC_FAT | GO:0031974~  membrane-enclosed lumen | Upregulation | 6.09E-05 | 117 | *NCBP1, MRPL42, STK38, DBF4, MORF4L2, CCT2, WTAP, CSNK2A1, PGRMC1, LRRC59, OGT, CCNA2, OXR1, MYO6, ANAPC5, ERP27, MAGOH, ENC1, PTBP1, PPP1CB, RFC5, MAPK1, MED17, VEGFA, XRN2, FUS, KIF4A, ME2, MCL1, NEK2, TRRAP, CALU, DDX3X, HNRNPF, CACYBP, SRGN, FN1, DDX42, FH, P4HB, ZMYM2, TSR1, RARS2, MKI67, SMAD4, CDC20, VDAC1, PPIF, NOLC1, SMARCC1, PCNA, DNAJB1, HSPD1, HNRNPH1, KIF23, EZH2, E2F8, DNAJC10, ZNF207, CCNE2, SAP30, DCAF13, FANCI, ZNF148, ZNF146, CDK12, TOP2A, TBL1XR1, CDK1, CDC6, SRPK2, RBBP4, CCNL1, TPX2, NUSAP1, MBD4, CDC5L, UBE2C, MCM4, PRPF4, NRIP1, MED6, IGF2R, CFL1, IPO5, KPNA2, MATR3, KPNA1, MED1, POLR2K, POLR2J, FKBP4, PRKDC, NFYB, EDEM3, MRPL13, SET, NUDT21, WAC, THBS1, BUB3, ACTB, POLR3K, CREB1, TRIM27, CENPF, BIRC5, STAT1, SMC3, CCNB1, PAPOLA, HSP90B1, ILF2, THRAP3, PSPC1, RCN2, KIF20A* |
| GOTERM_CC_FAT | GO:0043233~  organelle lumen | Upregulation | 5.69E-05 | 115 | *NCBP1, MRPL42, STK38, DBF4, MORF4L2, CCT2, WTAP, CSNK2A1, PGRMC1, LRRC59, OGT, CCNA2, OXR1, MYO6, ANAPC5, ERP27, MAGOH, ENC1, PTBP1, PPP1CB, RFC5, MAPK1, MED17, VEGFA, XRN2, FUS, KIF4A, ME2, MCL1, NEK2, TRRAP, CALU, DDX3X, HNRNPF, SRGN, FN1, DDX42, FH, P4HB, ZMYM2, TSR1, RARS2, MKI67, SMAD4, CDC20, VDAC1, PPIF, NOLC1, SMARCC1, PCNA, DNAJB1, HSPD1, HNRNPH1, KIF23, EZH2, E2F8, DNAJC10, ZNF207, CCNE2, SAP30, DCAF13, FANCI, ZNF148, ZNF146, CDK12, TOP2A, TBL1XR1, CDK1, CDC6, SRPK2, RBBP4, CCNL1, TPX2, NUSAP1, MBD4, CDC5L, UBE2C, MCM4, PRPF4, NRIP1, MED6, CFL1, IPO5, KPNA2, MATR3, KPNA1, MED1, POLR2K, POLR2J, FKBP4, PRKDC, NFYB, EDEM3, MRPL13, SET, NUDT21, WAC, THBS1, BUB3, ACTB, POLR3K, CREB1, TRIM27, CENPF, BIRC5, STAT1, SMC3, CCNB1, PAPOLA, HSP90B1, ILF2, THRAP3, PSPC1, RCN2, KIF20A* |
| GOTERM_CC_FAT | GO:0015630~  microtubule cytoskeleton | Upregulation | 6.84E-05 | 48 | *KIF23, PPP4R2, KIF4A, PRC1, NEK2, TTK, ARPC4, AURKA, CEP55, GTSE1, FAM83D, PSMB4, KIF2C, RANBP9, EZR, MACF1, NUDT21, BUB1, TOP2A, ASPM, KIF2A, CKAP2, KIF14, CDK1, CDC6, TBL1XR1, KIF11, CKAP5, DLGAP5, TPX2, CENPF, NUSAP1, CDC20, BIRC5, RACGAP1, SMC3, EML4, CCNB1, SS18, MAD2L1, CCNB2, SPAG5, PTP4A1, BUB1B, MAPRE1, ARL8B, TNFAIP3, KIF20A* |
| GOTERM_CC_FAT | GO:0031981~  nuclear lumen | Upregulation | 1.20E-04 | 95 | *KIF23, NCBP1, STK38, DBF4, MORF4L2, EZH2, E2F8, CCT2, WTAP, ZNF207, CCNE2, SAP30, DCAF13, CSNK2A1, FANCI, ZNF148, PGRMC1, ZNF146, CDK12, OGT, CCNA2, TOP2A, OXR1, SRPK2, TBL1XR1, CDC6, CDK1, RBBP4, MYO6, ANAPC5, MAGOH, PTBP1, ENC1, TPX2, CCNL1, NUSAP1, MBD4, CDC5L, UBE2C, MCM4, PPP1CB, PRPF4, NRIP1, MED6, RFC5, MAPK1, MED17, CFL1, IPO5, KPNA2, MATR3, XRN2, KPNA1, MED1, FUS, KIF4A, MCL1, POLR2K, NEK2, POLR2J, FKBP4, PRKDC, NFYB, TRRAP, SET, DDX3X, HNRNPF, NUDT21, WAC, BUB3, DDX42, ACTB, ZMYM2, TSR1, POLR3K, MKI67, CREB1, TRIM27, SMAD4, CENPF, CDC20, BIRC5, STAT1, SMC3, CCNB1, PAPOLA, ILF2, NOLC1, SMARCC1, THRAP3, PCNA, PSPC1, DNAJB1, HNRNPH1, KIF20A* |
| GOTERM_CC_FAT | GO:0070013~  intracellular organelle lumen | Upregulation | 1.14E-04 | 111 | *NCBP1, MRPL42, STK38, DBF4, MORF4L2, CCT2, WTAP, CSNK2A1, PGRMC1, LRRC59, OGT, CCNA2, OXR1, MYO6, ANAPC5, ERP27, MAGOH, ENC1, PTBP1, PPP1CB, RFC5, MAPK1, MED17, XRN2, FUS, KIF4A, ME2, MCL1, NEK2, TRRAP, CALU, DDX3X, HNRNPF, DDX42, FH, P4HB, ZMYM2, TSR1, RARS2, MKI67, SMAD4, CDC20, VDAC1, PPIF, NOLC1, SMARCC1, PCNA, DNAJB1, HSPD1, HNRNPH1, KIF23, EZH2, E2F8, DNAJC10, ZNF207, CCNE2, DCAF13, SAP30, FANCI, ZNF148, ZNF146, CDK12, TOP2A, TBL1XR1, CDK1, CDC6, SRPK2, RBBP4, CCNL1, TPX2, NUSAP1, MBD4, CDC5L, UBE2C, MCM4, PRPF4, NRIP1, MED6, CFL1, IPO5, KPNA2, MATR3, KPNA1, MED1, POLR2K, POLR2J, FKBP4, PRKDC, NFYB, EDEM3, MRPL13, SET, NUDT21, WAC, BUB3, ACTB, POLR3K, CREB1, TRIM27, CENPF, BIRC5, STAT1, SMC3, CCNB1, PAPOLA, HSP90B1, ILF2, THRAP3, PSPC1, RCN2, KIF20A* |
| GOTERM_CC_FAT | GO:0000776~  kinetochore | Upregulation | 1.11E-04 | 15 | *NUF2, CENPF, NDC80, CENPK, KIF2C, SUMO2, MAD2L1, PSEN1, CENPA, SPAG5, ZWINT, BUB1, BUB1B, ZWILCH, BUB3* |
| GOTERM_CC_FAT | GO:0005829~  cytosol | Upregulation | 1.56E-04 | 88 | *NCBP1, KYNU, CHMP4B, S100A7, PDLIM5, EIF5A, CCT2, FOXO3, PNP, CCNE2, CDC42, TDO2, PAK2, PIK3CA, OGT, RANBP2, FOXO3B, NET1, DLG1, CDC6, CDK1, MYO6, ANAPC5, NCALD, PIK3C2A, RAB4A, PRKCI, EIF2S3, UBE2C, ECT2, BCL2L11, MAPK1, ATP6V1A, GLUL, MAD2L1, RRM2, EIF4A2, RRM1, RAB14, BUB1B, MAPRE1, NFE2L2, SMS, KPNA1, LCP2, NEK2, COPZ1, FKBP1A, ABI1, KMO, MAPKAPK2, ATP6V1G1, ARFGEF2, HPRT1, TK1, GCH1, TYMS, EZR, SET, RAC1, HSPA8, BUB3, PLEC, ENO1, CSNK1A1, ACTB, VAV3, CKAP5, NAT1, GGH, SMAD4, EPRS, CDC20, ACLY, BIRC5, YWHAE, TAB2, PTPN12, CCNB1, HSP90B1, CCNB2, EIF4E, ARF1, PSMD12, PSMD11, PRKAR1A, RHEB, CPNE3, HSPD1* |
| GOTERM_CC_FAT | GO:0043232~  intracellular  non-membrane-bounded organelle | Upregulation | 4.23E-04 | 146 | *HIST2H2AA3, MRPL42, PRPF4B, PRC1, PDLIM5, MORF4L2, TTK, CCT2, AURKA, WTAP, PNP, CITED2, RAD21, CSNK2A1, H2AFV, HIST1H2BK, PGRMC1, HIST1H2BI, LRRC59, H2AFZ, H2AFY, ASPM, OXR1, TWF1, MYO6, ENC1, PTBP1, CTNNA1, RFC5, MAPK1, MAD2L1, BAZ1A, SPAG5, ZWINT, ARL8B, TNFAIP3, XRN2, FUS, KIF4A, NEK2, ARPC4, ANLN, ARPC5, PSMB4, MACF1, HNRNPF, HELLS, ARHGDIB, CKAP2, TSR1, MKI67, CKAP5, NUF2, NDC80, CDC20, VDAC1, NOLC1, SMARCC1, PCNA, HIST1H3D, DNAJB1, HNRNPH1, KIF23, GTSE1, ZNF207, ACTR3, FAM83D, ACTR2, DCAF13, KIF2C, RANBP9, H2BFS, PAK2, FANCI, ZNF148, ZNF146, RHOA, TOP2A, IFNGR1, DLG1, KIF2A, KIF14, CDK1, CDC6, TBL1XR1, SRPK2, RBBP4, KIF11, TPX2, NUSAP1, MBD4, CDC5L, MCM4, UBE2B, EML4, SS18, PSEN1, HIST2H2BE, KRIT1, CFL1, IPO5, BUB1B, MAPRE1, PPP4R2, YWHAZ, CNN3, FKBP4, CALD1, ABI1, CDH1, CEP55, TPM4, TPM3, SUMO2, MRPL13, EZR, CENPA, NUDT21, BUB1, ZWILCH, BUB3, PLEC, ACTB, MSH6, HIST1H2BD, EPPK1, HIST1H2BF, CREB1, HIST1H2BG, DLGAP5, HIST1H2BH, CENPF, BIRC5, RACGAP1, STAT1, CENPK, SMC2, SMC3, SMC4, CCNB1, PAPOLA, CCNB2, ILF2, PTP4A1, THRAP3, PSPC1, JAK1, KIF20A* |
| GOTERM_CC_FAT | GO:0043228~  non-membrane-bounded organelle | Upregulation | 4.23E-04 | 146 | *HIST2H2AA3, MRPL42, PRPF4B, PRC1, PDLIM5, MORF4L2, TTK, CCT2, AURKA, WTAP, PNP, CITED2, RAD21, CSNK2A1, H2AFV, HIST1H2BK, PGRMC1, HIST1H2BI, LRRC59, H2AFZ, H2AFY, ASPM, OXR1, TWF1, MYO6, ENC1, PTBP1, CTNNA1, RFC5, MAPK1, MAD2L1, BAZ1A, SPAG5, ZWINT, ARL8B, TNFAIP3, XRN2, FUS, KIF4A, NEK2, ARPC4, ANLN, ARPC5, PSMB4, MACF1, HNRNPF, HELLS, ARHGDIB, CKAP2, TSR1, MKI67, CKAP5, NUF2, NDC80, CDC20, VDAC1, NOLC1, SMARCC1, PCNA, HIST1H3D, DNAJB1, HNRNPH1, KIF23, GTSE1, ZNF207, ACTR3, FAM83D, ACTR2, DCAF13, KIF2C, RANBP9, H2BFS, PAK2, FANCI, ZNF148, ZNF146, RHOA, TOP2A, IFNGR1, DLG1, KIF2A, KIF14, CDK1, CDC6, TBL1XR1, SRPK2, RBBP4, KIF11, TPX2, NUSAP1, MBD4, CDC5L, MCM4, UBE2B, EML4, SS18, PSEN1, HIST2H2BE, KRIT1, CFL1, IPO5, BUB1B, MAPRE1, PPP4R2, YWHAZ, CNN3, FKBP4, CALD1, ABI1, CDH1, CEP55, TPM4, TPM3, SUMO2, MRPL13, EZR, CENPA, NUDT21, BUB1, ZWILCH, BUB3, PLEC, ACTB, MSH6, HIST1H2BD, EPPK1, HIST1H2BF, CREB1, HIST1H2BG, DLGAP5, HIST1H2BH, CENPF, BIRC5, RACGAP1, STAT1, CENPK, SMC2, SMC3, SMC4, CCNB1, PAPOLA, CCNB2, ILF2, PTP4A1, THRAP3, PSPC1, JAK1, KIF20A* |
| GOTERM_CC_FAT | GO:0000775~  chromosome, centromeric region | Upregulation | 3.90E-04 | 18 | *MKI67, NUF2, CENPF, NDC80, BIRC5, CENPK, KIF2C, SUMO2, MAD2L1, PSEN1, CENPA, SPAG5, ZWINT, BUB1, BUB1B, ZWILCH, HELLS, BUB3* |
| GOTERM_CC_FAT | GO:0005654~  nucleoplasm | Upregulation | 4.40E-04 | 63 | *KIF23, NCBP1, STK38, DBF4, EZH2, E2F8, WTAP, CCNE2, SAP30, CSNK2A1, FANCI, CDK12, OGT, TOP2A, CCNA2, CDC6, CDK1, TBL1XR1, RBBP4, MYO6, ANAPC5, MAGOH, PTBP1, CCNL1, CDC5L, UBE2C, MCM4, PRPF4, PPP1CB, NRIP1, MED6, RFC5, MAPK1, MED17, KPNA2, KPNA1, MED1, MCL1, POLR2K, POLR2J, PRKDC, NFYB, TRRAP, SET, DDX3X, HNRNPF, NUDT21, WAC, DDX42, ACTB, ZMYM2, POLR3K, CREB1, TRIM27, SMAD4, CDC20, CCNB1, SMARCC1, THRAP3, PSPC1, PCNA, HNRNPH1, KIF20A* |
| GOTERM_CC_FAT | GO:0048770~  pigment granule | Upregulation | 4.14E-04 | 15 | *HSP90AB1, P4HB, YWHAZ, GGH, ITGB1, YWHAE, CANX, CALU, LAMP1, HSP90B1, TFRC, RAC1, RAB5A, CTSB, HSPA8* |
| GOTERM_CC_FAT | GO:0042470~  melanosome | Upregulation | 4.14E-04 | 15 | *HSP90AB1, P4HB, YWHAZ, GGH, ITGB1, YWHAE, CANX, CALU, LAMP1, HSP90B1, TFRC, RAC1, RAB5A, CTSB, HSPA8* |
| GOTERM_CC_FAT | GO:0000793~  condensed chromosome | Upregulation | 5.31E-04 | 18 | *MKI67, NEK2, NUF2, CENPF, NDC80, CENPK, SMC2, SMC3, SMC4, KIF2C, MAD2L1, SPAG5, CENPA, ZWINT, BUB1, BUB1B, H2AFY, ZWILCH* |
| GOTERM_CC_FAT | GO:0000777~  condensed chromosome kinetochore | Upregulation | 5.10E-04 | 12 | *KIF2C, MAD2L1, SPAG5, CENPA, ZWINT, NUF2, BUB1, BUB1B, CENPF, NDC80, CENPK, ZWILCH* |
| GOTERM_CC_FAT | GO:0005783~  endoplasmic reticulum | Upregulation | 6.21E-04 | 66 | *S100A7, HMGCR, DNAJC10, EIF5A, TPD52, CANX, RAB1A, SSR1, PLOD2, PGRMC1, LRRC59, ANP32A, PSENEN, DNAJC3, KDELR1, IFNGR1, DLG1, KDELR3, KDELR2, SGPL1, SPTLC2, PIGX, ERP27, UGCG, UBE2J1, CNPY3, MAGT1, PSEN1, SQLE, VAMP7, RAB14, CTSC, DEGS1, HLA-DRA, OSTC, DERL1, UBE2V1, RSAD2, OAS1, ABI1, FKBP1A, HSPA1B, OAS2, EDEM3, GPRC5A, CALU, MIA3, SET, ALOX5AP, HSD17B6, PCMT1, SLC39A6, TRAM1, SEC61A1, SOAT1, P4HB, NCEH1, HSP90B1, SULF2, PTP4A1, CNIH4, SULF1, YIPF5, UBXN4, ALG13, RCN2* |
| GOTERM_CC_FAT | GO:0000786~  nucleosome | Upregulation | 9.67E-04 | 12 | *HIST2H2AA3, HIST1H2BD, HIST1H2BF, HIST1H2BG, HIST1H2BH, H2BFS, H2AFV, HIST1H2BK, CENPA, HIST2H2BE, HIST1H2BI, H2AFZ, H2AFY, HIST1H3D* |
| GOTERM_CC_FAT | GO:0044421~  extracellular region part | Downregulation | 2.16E-07 | 32 | *TF, LEPR, IGFBP6, CLU, CX3CL1, KIT, CCL28, OGN, LAMB3, APOD, SAA2, PTN, CFD, DPT, GHR, ANGPTL4, MATN2, LPL, TNXB, TNXA, EFEMP1, IGF1, ADIPOQ, VWF, CCL14, CXCL14, SFRP1, SCGB1D2, TGFBR3, SCGB3A1, DST, ADAMTS5, LIPE* |
| GOTERM_CC_FAT | GO:0005576~  extracellular region | Downregulation | 9.42E-06 | 44 | *TF, ENPP2, LEPR, IGFBP6, CLU, CX3CL1, KIT, CCL28, OGN, LAMB3, SAA2, APOD, PIP, PTN, ITIH5, CFD, PI15, DPT, GHR, ANGPTL4, MATN2, LPL, TNXB, TNXA, EFEMP1, LIFR, IGF1, PIGR, ADIPOQ, PLAC9, MUCL1, VWF, CHRDL1, CCL14, SFRP1, CXCL14, CLEC3B, SCGB1D2, TGFBR3, FCGBP, SCGB3A1, DST, SCGB2A2, ADAMTS5, LIPE* |
| GOTERM_CC_FAT | GO:0009986~  cell surface | Downregulation | 0.0173345 | 12 | *VWF, CAV1, SRPX, CD36, CRYAB, DMD, NTRK2, TGFBR3, KIT, CX3CL1, GHR, AOC3* |
| GOTERM_CC_FAT | GO:0031012~  extracellular matrix | Downregulation | 0.0193242 | 12 | *MATN2, LPL, TF, TNXB, TNXA, EFEMP1, OGN, VWF, LAMB3, DST, ADAMTS5, ANGPTL4, DPT* |
| GOTERM_CC_FAT | GO:0044449~ contractile fiber part | Downregulation | 0.0193896 | 7 | *JUP, ACTG2, ANK2, CRYAB, DMD, MYH11, SYNM* |
