# Supplementary material for: An in-silico method leads to recognition of hub genes and crucial pathways in survival of patients with breast cancer
Source: Sci Rep. 2020 Oct 30;10:18770. doi: 10.1038/s41598-020-76024-2 (PMC7603345; doi:10.1038/s41598-020-76024-2)
Supplement: Supplementary file 7 — Supplementary Information 7. [file 41598_2020_76024_MOESM7_ESM.docx]

An in-silico method leads to recognition of hub genes and crucial pathways in survival of patients with breast cancer

Sepideh Dashti^1^, Mohammad Taheri^2^, Soudeh Ghafouri-Fard^1^*

1. Department of Medical Genetics, Shahid Beheshti University of Medical Sciences, Tehran, Iran
2. Urogenital Stem Cell Research Center, Shahid Beheshti University of Medical Sciences, Tehran, Iran

Table S6. The result of GO enrichment analysis (GO for DEGs, Molecular function).

| **Category** | **Term** | **SampleGroup** | **Qvalue** | **Count** | **Genes** |
| --- | --- | --- | --- | --- | --- |
| GOTERM_MF_FAT | GO:0000166~  nucleotide binding | Upregulation | 6.08E-10 | 152 | *PRPF4B, STK38, HMGCR, RBM6, TTK, CCT2, AURKA, RNF213, RAB1A, NONO, CSNK2A1, TIA1, PIK3CA, MYO6, PTBP1, SPAG1, UBE2J1, MTPAP, EIF2S3, RFC5, MAPK1, MAP4K5, GLUL, RAB18, RIOK3, ASCC3, RAB14, ARL8B, RAB10, PRPS2, HLA-DRA, FUS, KIF4A, ME2, GNAI3, NEK2, ERBB2, OAS3, UBA6, OAS1, KMO, OAS2, HSPA1B, MAPKAPK2, UHMK1, CMPK2, TK1, DDX3X, GFM1, HNRNPF, RAC1, HNRNPC, SKIL, DYRK2, HELLS, DDX42, MKI67, RARS2, EPRS, ATAD2, SMG1, DDX58, NOLC1, ARF1, PRKAR1A, ARF4, RHEB, RIT1, HSPD1, PIP4K2A, HNRNPH1, HSP90AB1, KIF23, UBE2G1, CPEB4, DICER1, ACTR3, CDC42, ACTR2, HSPH1, KIF2C, PAK2, ATP8B1, CDK12, RHOA, RHOB, TOP2A, KIF2A, KIF14, ARL1, CDK1, CDC6, SRPK2, KIF11, PIK3C2A, RAB4A, PRKCI, TPX2, G3BP2, PBK, UBE2H, UBE2C, MCM4, UBE2B, UBE2N, ATP6V1A, TAF15, SQLE, EIF4A2, UBE2M, RRM1, RAB5A, ESRP1, BUB1B, UBE2S, MATR3, UBE2T, MELK, ALDH18A1, FKBP4, PRKDC, GCH1, TYMS, BUB1, DHX15, MSI2, ETNK1, HSPA4, GAPDH, HSPA8, TRIP13, ACTB, CSNK1A1, MSH6, MAT2A, MSH2, ACLY, SMC2, SMC3, SMC4, NRAS, RAB31, PAPOLA, HSP90B1, ILF2, GSPT1, THRAP3, PSPC1, JAK1, PGK1, PAICS, KIF20A* |
| GOTERM_MF_FAT | GO:0032555~  purine ribonucleotide binding | Upregulation | 3.04E-09 | 129 | *PRPF4B, STK38, TTK, CCT2, AURKA, RAB1A, CSNK2A1, PIK3CA, MYO6, SPAG1, UBE2J1, MTPAP, EIF2S3, RFC5, MAPK1, MAP4K5, GLUL, RAB18, ASCC3, RIOK3, RAB14, ARL8B, RAB10, PRPS2, HLA-DRA, KIF4A, GNAI3, NEK2, ERBB2, OAS3, UBA6, OAS1, OAS2, HSPA1B, MAPKAPK2, UHMK1, CMPK2, TK1, DDX3X, GFM1, RAC1, DYRK2, HELLS, DDX42, RARS2, MKI67, EPRS, ATAD2, SMG1, DDX58, NOLC1, ARF1, PRKAR1A, ARF4, RHEB, RIT1, HSPD1, PIP4K2A, HSP90AB1, KIF23, UBE2G1, DICER1, ACTR3, CDC42, ACTR2, HSPH1, KIF2C, PAK2, ATP8B1, CDK12, RHOA, RHOB, TOP2A, KIF2A, KIF14, CDC6, CDK1, SRPK2, ARL1, KIF11, PIK3C2A, RAB4A, PRKCI, TPX2, PBK, UBE2H, UBE2C, MCM4, UBE2B, UBE2N, ATP6V1A, EIF4A2, UBE2M, RRM1, RAB5A, BUB1B, UBE2S, MELK, UBE2T, ALDH18A1, FKBP4, PRKDC, GCH1, BUB1, DHX15, ETNK1, HSPA4, HSPA8, TRIP13, ACTB, CSNK1A1, MSH6, MAT2A, MSH2, ACLY, SMC2, SMC3, SMC4, NRAS, RAB31, PAPOLA, HSP90B1, ILF2, GSPT1, THRAP3, JAK1, PGK1, PAICS, KIF20A* |
| GOTERM_MF_FAT | GO:0032553~  ribonucleotide binding | Upregulation | 3.04E-09 | 129 | *PRPF4B, STK38, TTK, CCT2, AURKA, RAB1A, CSNK2A1, PIK3CA, MYO6, SPAG1, UBE2J1, MTPAP, EIF2S3, RFC5, MAPK1, MAP4K5, GLUL, RAB18, ASCC3, RIOK3, RAB14, ARL8B, RAB10, PRPS2, HLA-DRA, KIF4A, GNAI3, NEK2, ERBB2, OAS3, UBA6, OAS1, OAS2, HSPA1B, MAPKAPK2, UHMK1, CMPK2, TK1, DDX3X, GFM1, RAC1, DYRK2, HELLS, DDX42, RARS2, MKI67, EPRS, ATAD2, SMG1, DDX58, NOLC1, ARF1, PRKAR1A, ARF4, RHEB, RIT1, HSPD1, PIP4K2A, HSP90AB1, KIF23, UBE2G1, DICER1, ACTR3, CDC42, ACTR2, HSPH1, KIF2C, PAK2, ATP8B1, CDK12, RHOA, RHOB, TOP2A, KIF2A, KIF14, CDC6, CDK1, SRPK2, ARL1, KIF11, PIK3C2A, RAB4A, PRKCI, TPX2, PBK, UBE2H, UBE2C, MCM4, UBE2B, UBE2N, ATP6V1A, EIF4A2, UBE2M, RRM1, RAB5A, BUB1B, UBE2S, MELK, UBE2T, ALDH18A1, FKBP4, PRKDC, GCH1, BUB1, DHX15, ETNK1, HSPA4, HSPA8, TRIP13, ACTB, CSNK1A1, MSH6, MAT2A, MSH2, ACLY, SMC2, SMC3, SMC4, NRAS, RAB31, PAPOLA, HSP90B1, ILF2, GSPT1, THRAP3, JAK1, PGK1, PAICS, KIF20A* |
| GOTERM_MF_FAT | GO:0017076~  purine nucleotide binding | Upregulation | 9.28E-09 | 131 | *PRPF4B, STK38, TTK, CCT2, AURKA, RAB1A, CSNK2A1, PIK3CA, MYO6, SPAG1, UBE2J1, MTPAP, EIF2S3, RFC5, MAPK1, MAP4K5, GLUL, RAB18, ASCC3, RIOK3, RAB14, ARL8B, RAB10, PRPS2, HLA-DRA, KIF4A, GNAI3, NEK2, ERBB2, OAS3, UBA6, OAS1, KMO, OAS2, HSPA1B, MAPKAPK2, UHMK1, CMPK2, TK1, DDX3X, GFM1, RAC1, DYRK2, HELLS, DDX42, RARS2, MKI67, EPRS, ATAD2, SMG1, DDX58, NOLC1, ARF1, PRKAR1A, ARF4, RHEB, RIT1, HSPD1, PIP4K2A, HSP90AB1, KIF23, UBE2G1, DICER1, ACTR3, CDC42, ACTR2, HSPH1, KIF2C, PAK2, ATP8B1, CDK12, RHOA, RHOB, TOP2A, KIF2A, KIF14, CDC6, CDK1, SRPK2, ARL1, KIF11, PIK3C2A, RAB4A, PRKCI, TPX2, PBK, UBE2H, UBE2C, MCM4, UBE2B, UBE2N, ATP6V1A, SQLE, EIF4A2, UBE2M, RRM1, RAB5A, BUB1B, UBE2S, UBE2T, MELK, ALDH18A1, FKBP4, PRKDC, GCH1, BUB1, DHX15, ETNK1, HSPA4, HSPA8, TRIP13, ACTB, CSNK1A1, MSH6, MAT2A, MSH2, ACLY, SMC2, SMC3, SMC4, NRAS, RAB31, PAPOLA, HSP90B1, ILF2, GSPT1, THRAP3, JAK1, PGK1, PAICS, KIF20A* |
| GOTERM_MF_FAT | GO:0005524~  ATP binding | Upregulation | 5.57E-08 | 106 | *PRPF4B, STK38, TTK, CCT2, AURKA, RAB1A, CSNK2A1, PIK3CA, MYO6, UBE2J1, MTPAP, RFC5, MAPK1, MAP4K5, GLUL, RAB18, ASCC3, RIOK3, PRPS2, HLA-DRA, KIF4A, NEK2, ERBB2, OAS3, UBA6, OAS1, HSPA1B, OAS2, MAPKAPK2, UHMK1, TK1, CMPK2, DDX3X, DYRK2, HELLS, DDX42, RARS2, MKI67, EPRS, ATAD2, SMG1, DDX58, NOLC1, HSPD1, PIP4K2A, KIF23, HSP90AB1, UBE2G1, DICER1, ACTR3, HSPH1, KIF2C, ACTR2, PAK2, CDK12, ATP8B1, TOP2A, KIF2A, KIF14, CDC6, CDK1, SRPK2, KIF11, PIK3C2A, TPX2, PRKCI, PBK, UBE2H, UBE2C, MCM4, UBE2B, UBE2N, ATP6V1A, EIF4A2, UBE2M, RRM1, BUB1B, UBE2S, MELK, UBE2T, ALDH18A1, FKBP4, PRKDC, DHX15, BUB1, ETNK1, HSPA4, HSPA8, TRIP13, CSNK1A1, ACTB, MSH6, MAT2A, MSH2, ACLY, SMC2, SMC3, SMC4, PAPOLA, HSP90B1, ILF2, THRAP3, JAK1, PGK1, PAICS, KIF20A* |
| GOTERM_MF_FAT | GO:0032559~  adenyl ribonucleotide binding | Upregulation | 6.94E-08 | 107 | *PRPF4B, STK38, TTK, CCT2, AURKA, RAB1A, CSNK2A1, PIK3CA, MYO6, UBE2J1, MTPAP, RFC5, MAPK1, MAP4K5, GLUL, RAB18, ASCC3, RIOK3, PRPS2, HLA-DRA, KIF4A, NEK2, ERBB2, OAS3, UBA6, OAS1, HSPA1B, OAS2, MAPKAPK2, UHMK1, TK1, CMPK2, DDX3X, DYRK2, HELLS, DDX42, RARS2, MKI67, EPRS, ATAD2, SMG1, DDX58, NOLC1, PRKAR1A, HSPD1, PIP4K2A, KIF23, HSP90AB1, UBE2G1, DICER1, ACTR3, HSPH1, KIF2C, ACTR2, PAK2, CDK12, ATP8B1, TOP2A, KIF2A, KIF14, CDC6, CDK1, SRPK2, KIF11, PIK3C2A, TPX2, PRKCI, PBK, UBE2H, UBE2C, MCM4, UBE2B, UBE2N, ATP6V1A, EIF4A2, UBE2M, RRM1, BUB1B, UBE2S, MELK, UBE2T, ALDH18A1, FKBP4, PRKDC, BUB1, DHX15, ETNK1, HSPA4, HSPA8, TRIP13, CSNK1A1, ACTB, MSH6, MAT2A, MSH2, ACLY, SMC2, SMC3, SMC4, PAPOLA, HSP90B1, ILF2, THRAP3, JAK1, PGK1, PAICS, KIF20A* |
| GOTERM_MF_FAT | GO:0001882~  nucleoside binding | Upregulation | 8.36E-08 | 112 | *PRPF4B, STK38, TTK, CCT2, AURKA, PNP, RAB1A, CSNK2A1, PIK3CA, MYO6, UBE2J1, MTPAP, RFC5, MAPK1, MAP4K5, GLUL, RAB18, ASCC3, RIOK3, ARL8B, PRPS2, HLA-DRA, KIF4A, NEK2, ERBB2, OAS3, UBA6, OAS1, KMO, HSPA1B, OAS2, MAPKAPK2, UHMK1, TK1, CMPK2, DDX3X, DYRK2, HELLS, DDX42, RARS2, MKI67, EPRS, ATAD2, SMG1, DDX58, NOLC1, PRKAR1A, HSPD1, PIP4K2A, HSP90AB1, KIF23, UBE2G1, DICER1, ACTR3, ACTR2, HSPH1, KIF2C, PAK2, CDK12, ATP8B1, RHOB, TOP2A, KIF2A, KIF14, CDC6, CDK1, SRPK2, KIF11, PIK3C2A, TPX2, PRKCI, PBK, UBE2H, UBE2C, MCM4, UBE2B, UBE2N, ATP6V1A, SQLE, EIF4A2, UBE2M, RRM1, BUB1B, UBE2S, MELK, UBE2T, ALDH18A1, FKBP4, PRKDC, BUB1, DHX15, ETNK1, HSPA4, HSPA8, TRIP13, ACTB, CSNK1A1, MSH6, MAT2A, MSH2, ACLY, SMC2, SMC3, SMC4, PAPOLA, HSP90B1, ILF2, THRAP3, JAK1, PGK1, PAICS, KIF20A* |
| GOTERM_MF_FAT | GO:0001883~  purine nucleoside binding | Upregulation | 9.87E-08 | 111 | *PRPF4B, STK38, TTK, CCT2, AURKA, RAB1A, CSNK2A1, PIK3CA, MYO6, UBE2J1, MTPAP, RFC5, MAPK1, MAP4K5, GLUL, RAB18, ASCC3, RIOK3, ARL8B, PRPS2, HLA-DRA, KIF4A, NEK2, ERBB2, OAS3, UBA6, OAS1, KMO, HSPA1B, OAS2, MAPKAPK2, UHMK1, TK1, CMPK2, DDX3X, DYRK2, HELLS, DDX42, RARS2, MKI67, EPRS, ATAD2, SMG1, DDX58, NOLC1, PRKAR1A, HSPD1, PIP4K2A, HSP90AB1, KIF23, UBE2G1, DICER1, ACTR3, ACTR2, HSPH1, KIF2C, PAK2, CDK12, ATP8B1, RHOB, TOP2A, KIF2A, KIF14, CDC6, CDK1, SRPK2, KIF11, PIK3C2A, TPX2, PRKCI, PBK, UBE2H, UBE2C, MCM4, UBE2B, UBE2N, ATP6V1A, SQLE, EIF4A2, UBE2M, RRM1, BUB1B, UBE2S, MELK, UBE2T, ALDH18A1, FKBP4, PRKDC, BUB1, DHX15, ETNK1, HSPA4, HSPA8, TRIP13, ACTB, CSNK1A1, MSH6, MAT2A, MSH2, ACLY, SMC2, SMC3, SMC4, PAPOLA, HSP90B1, ILF2, THRAP3, JAK1, PGK1, PAICS, KIF20A* |
| GOTERM_MF_FAT | GO:0030554~  adenyl nucleotide binding | Upregulation | 1.58E-07 | 109 | *PRPF4B, STK38, TTK, CCT2, AURKA, RAB1A, CSNK2A1, PIK3CA, MYO6, UBE2J1, MTPAP, RFC5, MAPK1, MAP4K5, GLUL, RAB18, ASCC3, RIOK3, PRPS2, HLA-DRA, KIF4A, NEK2, ERBB2, OAS3, UBA6, OAS1, KMO, HSPA1B, OAS2, MAPKAPK2, UHMK1, TK1, CMPK2, DDX3X, DYRK2, HELLS, DDX42, RARS2, MKI67, EPRS, ATAD2, SMG1, DDX58, NOLC1, PRKAR1A, HSPD1, PIP4K2A, HSP90AB1, KIF23, UBE2G1, DICER1, ACTR3, HSPH1, KIF2C, ACTR2, PAK2, CDK12, ATP8B1, TOP2A, KIF2A, KIF14, CDC6, CDK1, SRPK2, KIF11, PIK3C2A, TPX2, PRKCI, PBK, UBE2H, UBE2C, MCM4, UBE2B, UBE2N, ATP6V1A, SQLE, EIF4A2, UBE2M, RRM1, BUB1B, UBE2S, MELK, UBE2T, ALDH18A1, FKBP4, PRKDC, BUB1, DHX15, ETNK1, HSPA4, HSPA8, TRIP13, CSNK1A1, ACTB, MSH6, MAT2A, MSH2, ACLY, SMC2, SMC3, SMC4, PAPOLA, HSP90B1, ILF2, THRAP3, JAK1, PGK1, PAICS, KIF20A* |
| GOTERM_MF_FAT | GO:0003924~  GTPase activity | Upregulation | 0.0058934 | 22 | *ARL1, GNAI3, RAB4A, EIF2S3, RAB1A, CDC42, NRAS, RAB31, ARF1, RAB18, GSPT1, GNB1, GFM1, RAC1, ARF4, RHOA, RAB5A, RAB14, RHEB, RHOB, RIT1, ARL8B* |
| GOTERM_MF_FAT | GO:0001948~  glycoprotein binding | Upregulation | 0.0061173 | 9 | *PTPRC, ERBB2, IGF2R, COMP, RAB14, ITGB2, THBS1, COL5A1, RASA1* |
| GOTERM_MF_FAT | GO:0003723~  RNA binding | Upregulation | 0.0398865 | 47 | *FUS, NCBP1, CPEB4, DICER1, ASCC1, OAS3, RBM6, EIF5A, OAS1, OAS2, UHMK1, APOBEC3B, NONO, ZFP36L2, DDX3X, TIA1, HNRNPF, NUDT21, MSI2, LSM4, RBM47, HNRNPC, DDX42, RAD51AP1, MAGOH, PTBP1, MTPAP, G3BP2, EPRS, MBNL1, CDC5L, LARP4B, SLBP, DDX58, PAPOLA, HSP90B1, EIF4E, TAF15, ILF2, FAM120A, EIF4A2, PSPC1, ESRP1, MEX3D, SRP72, HNRNPH1, MATR3* |
| GOTERM_MF_FAT | GO:0008092~  cytoskeletal protein binding | Downregulation | 0.0106029 | 16 | *CRYAB, ABLIM3, SYNPO2, JUP, TNS1, SORBS1, DMD, MYH11, PIP, OPHN1, SYNM, LMOD1, CNN1, DST, MYLK, PARVA* |
| GOTERM_MF_FAT | GO:0003779~actin binding | Downregulation | 0.0142574 | 12 | *TNS1, SORBS1, ABLIM3, DMD, MYH11, PIP, OPHN1, SYNPO2, CNN1, DST, MYLK, PARVA* |
| GOTERM_MF_FAT | GO:0005198~  structural molecule activity | Downregulation | 0.0201896 | 17 | *CLDN8, CAV1, NES, TNXB, TNXA, CRYAB, CLDN5, CLDN11, JUP, LAMB3, KRT17, KRT5, DMD, KRT15, KRT14, MYH11, SYNM, RPS11* |
| GOTERM_MF_FAT | GO:0005200~  structural constituent of cytoskeleton | Downregulation | 0.0296933 | 6 | *KRT17, KRT5, DMD, KRT15, KRT14, SYNM* |
| GOTERM_MF_FAT | GO:0047115~  trans-1,2-dihydrobenzene-1,2-diol dehydrogenase activity | Downregulation | 0.0352961 | 3 | *AKR1C3, AKR1C2, AKR1C1* |
