# Supplementary material for: An in-silico method leads to recognition of hub genes and crucial pathways in survival of patients with breast cancer
Source: Sci Rep. 2020 Oct 30;10:18770. doi: 10.1038/s41598-020-76024-2 (PMC7603345; doi:10.1038/s41598-020-76024-2)
Supplement: Supplementary file 10 — Supplementary Information 10. [file 41598_2020_76024_MOESM10_ESM.docx]

An in-silico method leads to recognition of hub genes and crucial pathways in survival of patients with breast cancer

Sepideh Dashti^1^, Mohammad Taheri^2^, Soudeh Ghafouri-Fard^1^*

1. Department of Medical Genetics, Shahid Beheshti University of Medical Sciences, Tehran, Iran
2. Urogenital Stem Cell Research Center, Shahid Beheshti University of Medical Sciences, Tehran, Iran

Table S9. Pearson correlation coefficient for 20 candidate genes (p-value < 2.2e-16).

| **Correlation** | ***CDK1*** | ***CCNB1*** | ***CCNA2*** | ***CDC20*** | ***MAD2L1*** | ***KIF11*** | ***CENPA*** | ***PCNA*** | ***EZH2*** | ***KIF23*** | ***TOP2A*** | ***UBE2C*** | ***BIRC5*** | ***KIF2C*** | ***RRM2*** | ***RACGAP1*** | ***KIF4A*** | ***KPNA2*** | ***TYMS*** | ***RRM1*** |
| --- | --- | --- | --- | --- | --- | --- | --- | --- | --- | --- | --- | --- | --- | --- | --- | --- | --- | --- | --- | --- |
| ***CDK1*** | 1 | 0.897 | 0.85 | 0.85 | 0.89 | 0.92 | 0.9 | 0.8 | 0.84 | 0.859 | 0.8 | 0.9 | 0.81 | 0.852 | 0.903 | 0.8602 | 0.8568 | 0.83 | 0.8 | 0.782 |
| ***CCNB1*** | 0.9 | 1 | 0.868 | 0.88 | 0.9 | 0.9 | 0.9 | 0.8 | 0.86 | 0.888 | 0.8 | 0.9 | 0.83 | 0.866 | 0.902 | 0.9141 | 0.8909 | 0.86 | 0.8 | 0.706 |
| ***CCNA2*** | 0.85 | 0.868 | 1 | 0.9 | 0.94 | 0.87 | 0.9 | 0.8 | 0.85 | 0.888 | 0.8 | 0.9 | 0.85 | 0.895 | 0.837 | 0.8721 | 0.9204 | 0.8 | 0.8 | 0.652 |
| ***CDC20*** | 0.85 | 0.88 | 0.9 | 1 | 0.88 | 0.86 | 0.9 | 0.7 | 0.86 | 0.891 | 0.7 | 0.9 | 0.85 | 0.963 | 0.852 | 0.8746 | 0.929 | 0.79 | 0.9 | 0.614 |
| ***MAD2L1*** | 0.89 | 0.898 | 0.936 | 0.88 | 1 | 0.9 | 0.9 | 0.8 | 0.85 | 0.887 | 0.8 | 0.9 | 0.85 | 0.885 | 0.87 | 0.8937 | 0.8897 | 0.86 | 0.8 | 0.703 |
| ***KIF11*** | 0.92 | 0.902 | 0.869 | 0.86 | 0.9 | 1 | 0.9 | 0.8 | 0.87 | 0.877 | 0.8 | 0.9 | 0.83 | 0.845 | 0.897 | 0.8969 | 0.8823 | 0.82 | 0.8 | 0.708 |
| ***CENPA*** | 0.89 | 0.893 | 0.923 | 0.92 | 0.92 | 0.88 | 1 | 0.8 | 0.83 | 0.897 | 0.8 | 0.9 | 0.84 | 0.914 | 0.87 | 0.8828 | 0.9176 | 0.81 | 0.8 | 0.698 |
| ***PCNA*** | 0.81 | 0.788 | 0.754 | 0.71 | 0.81 | 0.83 | 0.8 | 1 | 0.79 | 0.79 | 0.8 | 0.8 | 0.69 | 0.722 | 0.778 | 0.7806 | 0.7246 | 0.79 | 0.8 | 0.753 |
| ***EZH2*** | 0.84 | 0.856 | 0.852 | 0.86 | 0.85 | 0.87 | 0.8 | 0.8 | 1 | 0.845 | 0.8 | 0.8 | 0.8 | 0.857 | 0.884 | 0.8697 | 0.8635 | 0.83 | 0.8 | 0.589 |
| ***KIF23*** | 0.86 | 0.888 | 0.888 | 0.89 | 0.89 | 0.88 | 0.9 | 0.8 | 0.85 | 1 | 0.8 | 0.9 | 0.81 | 0.871 | 0.843 | 0.8811 | 0.8785 | 0.83 | 0.8 | 0.684 |
| ***TOP2A*** | 0.78 | 0.819 | 0.752 | 0.75 | 0.81 | 0.83 | 0.8 | 0.8 | 0.77 | 0.792 | 1 | 0.8 | 0.78 | 0.735 | 0.802 | 0.8422 | 0.7669 | 0.79 | 0.8 | 0.59 |
| ***UBE2C*** | 0.91 | 0.924 | 0.889 | 0.89 | 0.91 | 0.9 | 0.9 | 0.8 | 0.85 | 0.878 | 0.8 | 1 | 0.87 | 0.891 | 0.884 | 0.9127 | 0.9071 | 0.85 | 0.8 | 0.676 |
| ***BIRC5*** | 0.81 | 0.834 | 0.85 | 0.85 | 0.85 | 0.83 | 0.8 | 0.7 | 0.8 | 0.809 | 0.8 | 0.9 | 1 | 0.854 | 0.819 | 0.8556 | 0.8615 | 0.8 | 0.8 | 0.568 |
| ***KIF2C*** | 0.85 | 0.866 | 0.895 | 0.96 | 0.89 | 0.84 | 0.9 | 0.7 | 0.86 | 0.871 | 0.7 | 0.9 | 0.85 | 1 | 0.847 | 0.8718 | 0.9144 | 0.81 | 0.9 | 0.635 |
| ***RRM2*** | 0.9 | 0.902 | 0.837 | 0.85 | 0.87 | 0.9 | 0.9 | 0.8 | 0.88 | 0.843 | 0.8 | 0.9 | 0.82 | 0.847 | 1 | 0.8902 | 0.864 | 0.85 | 0.8 | 0.686 |
| ***RACGAP1*** | 0.86 | 0.914 | 0.872 | 0.87 | 0.89 | 0.9 | 0.9 | 0.8 | 0.87 | 0.881 | 0.8 | 0.9 | 0.86 | 0.872 | 0.89 | 1 | 0.9071 | 0.83 | 0.8 | 0.622 |
| ***KIF4A*** | 0.86 | 0.891 | 0.92 | 0.93 | 0.89 | 0.88 | 0.9 | 0.7 | 0.86 | 0.878 | 0.8 | 0.9 | 0.86 | 0.914 | 0.864 | 0.9071 | 1 | 0.8 | 0.8 | 0.601 |
| ***KPNA2*** | 0.83 | 0.856 | 0.798 | 0.79 | 0.86 | 0.82 | 0.8 | 0.8 | 0.83 | 0.832 | 0.8 | 0.9 | 0.8 | 0.809 | 0.854 | 0.8282 | 0.7961 | 1 | 0.8 | 0.696 |
| ***TYMS*** | 0.83 | 0.805 | 0.845 | 0.86 | 0.85 | 0.85 | 0.8 | 0.8 | 0.84 | 0.84 | 0.8 | 0.8 | 0.75 | 0.851 | 0.802 | 0.8018 | 0.8325 | 0.77 | 1 | 0.655 |
| ***RRM1*** | 0.78 | 0.706 | 0.652 | 0.61 | 0.7 | 0.71 | 0.7 | 0.8 | 0.59 | 0.684 | 0.6 | 0.7 | 0.57 | 0.635 | 0.686 | 0.6218 | 0.6015 | 0.7 | 0.7 | 1 |
